# FGF9–FGFR2 Signaling via Osteocytes‐Preosteoblasts Crosstalks to Mediate Mechanotransduction‐Driven Intramembranous Osteogenesis in the Underdeveloped Maxilla

**DOI:** 10.1002/advs.202506954

**Published:** 2025-09-06

**Authors:** Yiwen Zhou, Lili Chen, Miaomiao Han, Peixiang Zhu, Yanyi Wang, Xuanxuan Yu, Tingyu Reng, Huijuan Wang, Baochao Li, Caixia Zhang, Ziwei Huang, Shuang Lin, Guoyun Wang, Jie Ge, Baosheng Guo, Huang Li

**Affiliations:** ^1^ Department of Orthodontics Nanjing Stomatological Hospital Affiliated Hospital of Medical School Institute of Stomatology Nanjing University 30 Zhongyang Road Nanjing Jiangsu 210008 China; ^2^ Department of Stomatology the First People's Hospital of Changzhou the Third Affiliated Hospital of Soochow University Changzhou 213003 China; ^3^ State Key Laboratory of Pharmaceutical Biotechnology Division of Sports Medicine and Adult Reconstructive Surgery Department of Orthopedic Surgery Affiliated Drum Tower Hospital Medical School Nanjing University Nanjing 210008 China

**Keywords:** fibroblast growth factor, maxillary underdevelopment, mechanical stress, osteocytes, preosteoblasts

## Abstract

Maxillary underdevelopment is a critical component of skeletal Class III malocclusion, closely linked to altered biomechanical signaling. Mechanical stimulation through early facemask protraction can effectively promote maxillary growth, yet the underlying mechanotransduction mechanisms remain unclear. In this study, fibroblast growth factor 9 (FGF9) is identified as a key biomechanical responder in maxillary development. Secreted predominantly by osteocytes, FGF9 interacts with fibroblast growth factor receptor 2 (FGFR2) on preosteoblasts to inhibit osteogenic differentiation. Mechanical stress reduces FGF9 secretion from osteocytes, thereby relieving its inhibitory effect and enhancing osteogenesis. Mechanistically, FGF9 promotes nuclear translocation of FGFR2 in preosteoblasts, which modulates transcription factors ATF5 and NR2F1 to suppress bone formation. In vivo, targeted overexpression of FGF9 in bone tissue led to significant maxillary growth impairment, underlying the pathological impact of disrupted mechanical signaling. These findings reveal a novel osteocyte–preosteoblast axis regulated by FGF9–FGFR2 signaling in response to mechanical stimulation and provide mechanistic insight into how biomechanical forces shape craniofacial development. This pathway offers new mechanistic insights and potential therapeutic targets for correcting craniofacial skeletal abnormalities.

## Graphical abstract

This study identifies osteocyte‐derived FGF9 as a mechanosensitive factor that impairs osteogenesis in the underdeveloped maxilla by inducing FGFR2 nuclear translocation in preosteoblasts. Mechanical stimulation reduces FGF9 secretion, relieving its inhibitory effect. These findings reveal a novel osteocyte–preosteoblast FGF9–FGFR2 axis in craniofacial mechanotransduction and offer new insights for therapeutic modulation of maxillary growth.

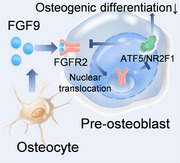

## 1 Introduction

Skeletal Class III malocclusion is a multifactorial and complex disease, characterized by maxillary underdevelopment, mandibular overdevelopment, or a combination of both.^[^ ^1^, ^2^ ^]^ Among the skeletal Class III population, 57% of the patients exhibited a deficiency in the maxilla.^[^ ^3^ ^]^ This maxillary underdevelopment is largely driven by genetic factors, with insufficient osteogenesis emerging as a critical pathological mechanism. Clinically, facemask protraction devices are widely employed during early orthodontic treatment to stimulate maxillary growth, promoting bone remodeling through mechanical stress.^[^ ^4^ ^]^ However, the precise mechanobiological pathways by which mechanical traction facilitates maxillary development remain to be fully elucidated.

Developed through intramembranous ossification, the sagittal growth of the maxilla originates from the maxillary tuberosity.^[^ ^5^ ^]^ During maxillary bone remodeling, several cells are involved, which are defined as basic multicellular unit (BMU).^[^ ^6^ ^]^The differentiation and mineralization of preosteoblasts within the BMU are mainly regulated by paracrine signals from neighboring cells.^[^ ^7^ ^]^ Notably, osteocytes—comprising ≈95% of all bone cells in the maxilla—are encapsulated within the mineralized bone matrix and serve as the primary mechanosensor,^[^ ^8^ ^]^ detecting mechanical stress and relaying signals to other effector cells.^[^ ^9^, ^10^ ^]^ However, the role of osteocytes as a signaling hub in regulating intercellular interactions and its specific contribution to underdeveloped maxillary under mechanical stress protraction has not been elucidated yet.

Fibroblast growth factor (FGF) signaling plays an essential role in maxillary development and in the pathogenesis of skeletal Class III malocclusion. Among these, fibroblast growth factor 9 (FGF9), a paracrine factor specifically highly expressed in osteocytes,^[^ ^11^, ^12^, ^13^ ^]^ plays a critical role in bone development by modulating both the proliferation and differentiation of cells within the BMU.^[^ ^14^, ^15^, ^16^, ^17^, ^18^, ^19^ ^]^ As a paracrine ligand, FGF9 exhibits high affinity with fibroblast growth factor receptor 2 (FGFR2), a membrane receptor predominantly expressed on preosteoblasts.^[^ ^20^ ^]^ In gene enrichment analysis of skeletal Class III malocclusion, fibroblast growth factor family (FGF‐FGFR) signals rank at the top.^[^ ^21^ ^]^ Meanwhile, our previous study has identified FGFR2 as a key factor associated with this malocclusion.^[^ ^22^ ^]^ Nevertheless, whether the intercellular communication between osteocytes and preosteoblasts via FGF9 and FGFR2 axis under mechanical stress regulates bone remodeling remains unknown.

In this study, we established a facemask protraction model in mice to investigate how mechanical stress influences maxillary development. Our findings first reveal that osteocyte‐secreted FGF9 plays a pivotal role in mediating bone remodeling under mechanical stress. Specifically, mechanical stress down‐regulated FGF9 expression in osteocytes, which in turn enhanced osteogenesis of preosteoblasts while inhibiting osteoclastogenesis, thereby promoting maxillary development. Mice with bone‐targeted adenoviral overexpression of FGF9 exhibited an underdeveloped maxilla and abnormal edge‐to‐edge malocclusion. Mechanistically, FGF9 impedes osteogenic differentiation of preosteoblasts by inducing the nuclear translocation of FGFR2 and modulating downstream transcription factors, including ATF5 and NR2F1. Finally, the overexpression of FGF9 was found in the patient with maxillary underdevelopment. In summary, our study uncovers a novel intercellular interaction between osteocytes and preosteoblasts mediated by the FGF9‐FGFR2 axis, which regulates maxillary growth under mechanical stress.

## 2 Results

### 2.1 Mechanical Stress Promotes Bone Formation in the Underdeveloped Maxilla and Down‐Regulates FGF9 Expression in Osteocytes

**Figure 1:**
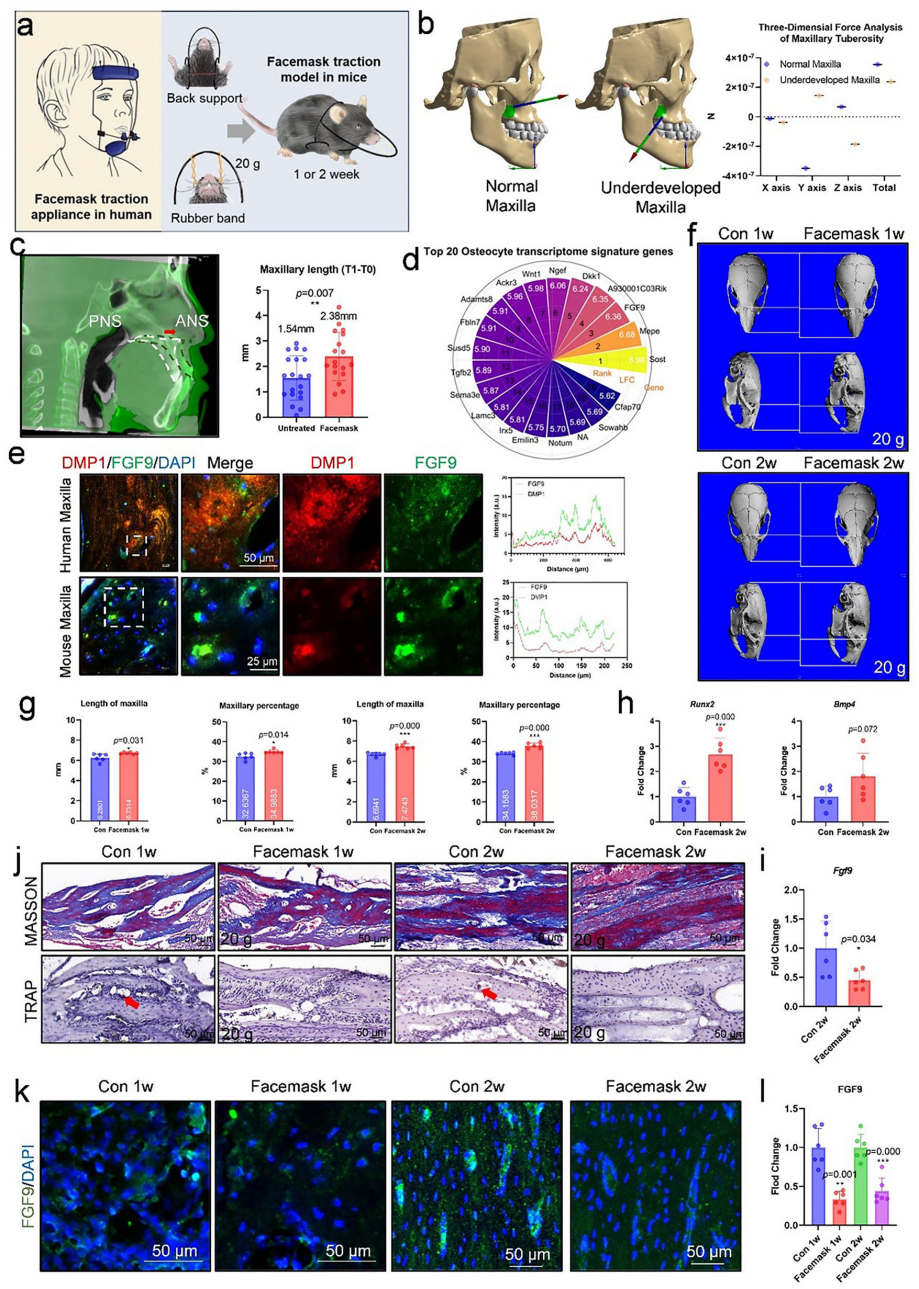
Mechanical stretch stress promoted maxillary development and reduced FGF9 expression in osteocytes. a) Schematic diagram of experiments in this section. b) The 3D analysis images of normal maxilla and underdeveloped maxilla under masticatory muscle strength and its quantification. c) Sagittal section of CBCT measuring the maxillary length (ANS‐PNS) of patients before and after facemask protraction and its maxillary growth quantification (untreated *n* = 20, facemask treated *n* = 18). d) Top 20 genes that were specifically expressed in osteocytes. e) Dural immunofluorescence images of DMP1 and FGF9 in human and mouse maxilla and its colocalization quantification. f,g) Micro‐CT reconstructed images of mice after facemask protraction for 1 or 2 week and quantification of maxillary length and maxillary percentage. h,i) The mRNA level of *Runx2*, *Bmp4*, and *Fgf9* in the maxilla of mice after 2‐week facemask protraction. j) The representative images of MASSON and TRAP staining of maxilla after facemask protraction. The red arrow represents TRAP staining regions. k,l) Representative immunofluorescence images and its quantification of FGF9 in the maxilla of mice after facemask protraction. Scale bar = 50 µm. Data are presented as mean ± SD. Statistical comparison between groups was performed using an independent samples *t*‐test. Statistical significance was determined as follows: *p* < 0.05*, *p* < 0.01 **, *p* < 0.001***. Facemask protraction *n* = 6.

To understand the mechanical stress distribution in the underdeveloped maxilla, we first performed computerized finite element analysis (FEA) to map the mechanical stress distribution. The 3D reconstructed images showed that, compared with the normal maxilla with upward and forward stress, the underdeveloped maxilla displayed a downward and backward stress and the stress level was significantly lower (**Figures** **1**b and S8, Supporting Information), indicating an insufficient and abnormal mechanical stress distribution in the underdeveloped maxilla.

Next, we detected the effect of facemask protraction on maxillary development in the clinic (Table S9 and Figure S2a, Supporting Information). Consistent with other studies,^[^ ^23^ ^]^ in adolescents, facemask protraction significantly promoted the maxillary growth, with increased maxillary length which was measured by ANS‐PNS distance (Figure 1c and Figure S2b, Supporting Information), demonstrating the positive effects of mechanical stress on bone remodeling.

Then, we identified that mechanical stress promoted maxillary growth via osteocytes, the primary mechanosensitive cells within bone which regulate osteoblast activity through paracrine factors.^[^ ^8^ ^]^ Among the osteocytes signature genes, fibroblast growth factor 9 (FGF9), a specific hypersecretion factor (Figure 1d), was highly expressed in both the embryo and postnatal stage during maxilla development (Figure S5a–c, Supporting Information) and expressed in bone lacuna co‐located with osteocytes’ specific marker DMP1 (Figure 1e; Figure S5e, Supporting Information).

In order to detect the role of FGF9 in the crosstalk between osteocytes and preosteoblasts after mechanical stimulation, we established the facemask protraction model (FM) in mice (Figure 1a; Figure S2c, Supporting Information). With 20 g mechanical stress on maxilla for 1 or 2 weeks, the length of maxilla and maxilla‐to‐skull ratio were effectively improved compared to the Sham group (Figure 1f,g) with nearly the same bone mass in the maxilla (Figure S2e, Supporting Information). There was more new bone formation in the FM group confirmed by Masson staining (Figure 1j; Figure S2h, Supporting Information). The mRNA and protein level of osteogenic markers such as RUNX2 and BMP4 also increased with mechanical stress (Figure 1h; Figure S2f,g, Supporting Information). In contrast, the staining patterns of Tartrate‐resistant acid phosphatase (TRAP) showed a decreased osteoclast activity (Figure 1j; Figure S2h, Supporting Information). The most important thing is, the decreased protein expression of FGF9 in osteocyte lacunae was found in FM group under mechanical stress (Figure 1k,l). The mRNA level of FGF9 in the maxilla was also significantly declined (Figure 1i). Additionally, the cell proliferation and apoptosis were not affected (Figure S2i,j, Supporting Information). These results validated that mechanical stress could promote maxillary growth and downregulate FGF9 expression.

### 2.2 In Vitro Mechanical Stress Down‐Regulates FGF9 Expression in Osteocytes and Subsequently Promotes Osteogenesis in Preosteoblasts

**Figure 2:**
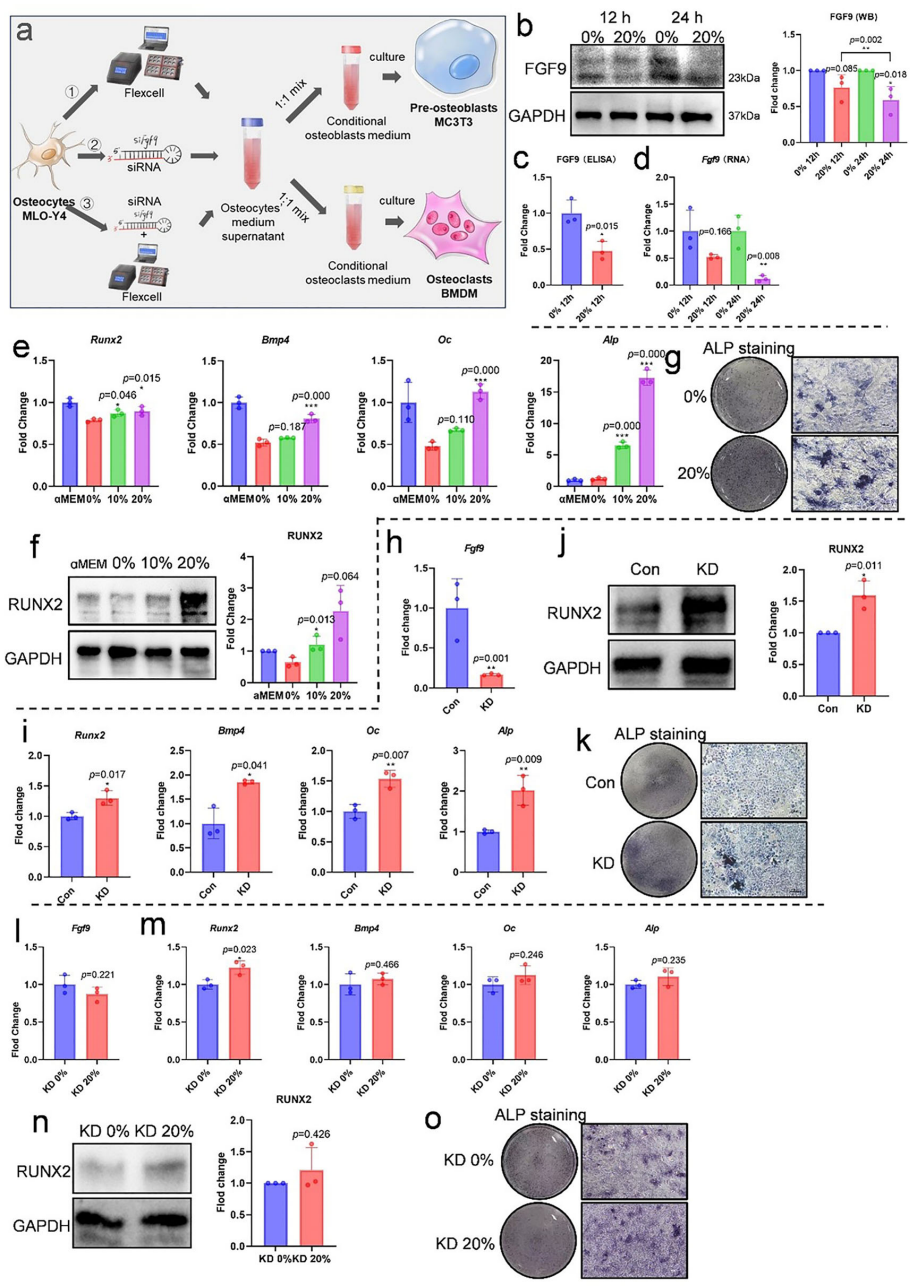
Mechanical stress reduced FGF9 expression in osteocytes and promoted osteogenesis in preosteoblasts. a) Schematic diagram of experiments in this section. b–d) The mRNA and protein level of FGF9 in osteocytes were quantified by qPCR, Western blot, and ELISA after applying 20% cyclic stretch for 12 or 24 h. e–g) Osteogenic differentiation of MC3T3 following co‐culture with osteocytes’ mechanical stress conditional medium: e) qPCR analysis of *Runx2, Bmp4, Oc*, and *Alp* mRNA expression; f) Representative immunoblot images and quantification of RUNX2 protein levels; g) Representative ALP staining images. h) The mRNA level of *Fgf9* in osteocytes after siRNA knockdown. i–k) Osteogenic differentiation of MC3T3 following co‐culture with osteocytes’ FGF9 knockdown conditional medium: i) qPCR analysis of *Runx2, Bmp4, Oc*, and *Alp* mRNA expression; j) Representative immunoblot images and quantification of RUNX2 protein levels; k) Representative ALP staining images. l) The mRNA level of *Fgf9* in knockdown osteocytes after 20% cyclic stretch stress. m–o) Osteogenic differentiation of MC3T3 following co‐culture with osteocytes’ FGF9 knockdown and mechanical stress conditional medium: m) qPCR analysis of *Runx2, Bmp4, Oc*, and *Alp* mRNA expression; n) Representative immunoblot images and quantification of RUNX2 protein levels; o) Representative ALP staining images. Scale bar = 100 µm. Data are presented as mean ± SD. *n* = 3. Statistical comparison between groups was performed using paired t‐test and independent samples t‐test. Statistical significance was determined as follows: *p* < 0.05*, *p* < 0.01 **, *p* < 0.001***.

To demonstrate FGF9 has a specific role between osteocytes and preosteoblasts, we applied in vitro cyclic stretch to osteocytes using a Flexcell system (**Figure** **2**a). A significant decrease in FGF9 expression was observed under 20% cyclic stretch for 12 h, as confirmed by qPCR and Western blot, and its reduced secretion was verified by ELISA (Figure 2b–d). Moreover, this reduction was even more pronounced with the longer duration of mechanical stress (24 h). Then, we conducted co‐culture experiments with preosteoblasts and osteoclasts (Figure 2a). Conditional media from osteocytes exposed to 10% or 20% mechanical stretch (10% CM, 20% CM) significantly enhanced osteogenesis in MC3T3 cells. Compared with the control group (0% CM), both 10% and 20% CM groups promoted osteoblastic differentiation and mineralization, which was demonstrated by up‐regulated osteogenic markers including *Runx2, Bmp4, Oc, and Alp* mRNA levels (Figure 2e), increased RUNX2 protein levels (Figure 2f), and elevated ALP activity (Figure 2g; Figure S7m, Supporting Information). In contrast, osteoclast differentiation was inhibited when cultured with 10% CM and 20% CM (Figure S7a–c,j, Supporting Information). Collectively, these results confirm that FGF9 secreted by osteocytes was reduced and subsequently promote bone remodeling through paracrine pathway under mechanical stress.

To further elucidate the role of FGF9, we used small interfering RNA (siRNA) to knock down FGF9 secretion in osteocytes (Figure 2h; Figure S6a,b, Supporting Information) and co‐cultured the knockdown conditional medium with preosteoblasts and osteoclasts (Figure 2a). The conditional medium from FGF9 knockdown osteocytes (KD CM) showed similar function as mechanical stress conditional medium. The expression of osteogenic markers in KD CM group increased, with higher ALP activity compared to the nonknockdown group (Con CM), while simultaneously inhibiting osteoclast differentiation (Figure 2i–k; Figure S7d–f,k,m, Supporting Information). These findings demonstrate that the reduction of FGF9 from osteocytes could promote osteogenesis.

To reverse validate FGF9's FGF9 pivotal role among the array of mechanical‐sensitive paracrine factors derived by osteocytes, we applied cyclic stretch stress to FGF9 knockdown osteocytes (KD 20%). Since FGF9 expression was already reduced by siRNA, the additional effect of mechanical force was minimal (Figure 2l), which can assess the contribution of other mechanosensitive factors besides FGF9. Compared to the KD 0% CM, the conditioned medium from KD 20% osteocytes (KD 20% CM) caused a lesser extent of osteoblast differentiation and mineralization and osteoclast differentiation than that observed between 20% CM and 0% CM (Figure 2m–o; Figure S7g–i,l,m, Supporting Information). To sum up, these results confirm that reduced FGF9 secretion plays a key role as a mechanosensitive factor in osteocytes, which significantly enhances bone formation.

### 2.3 Recombinant FGF9 Protein Inhibits Osteogenesis and Maxillary Bone Formation

**Figure 3:**
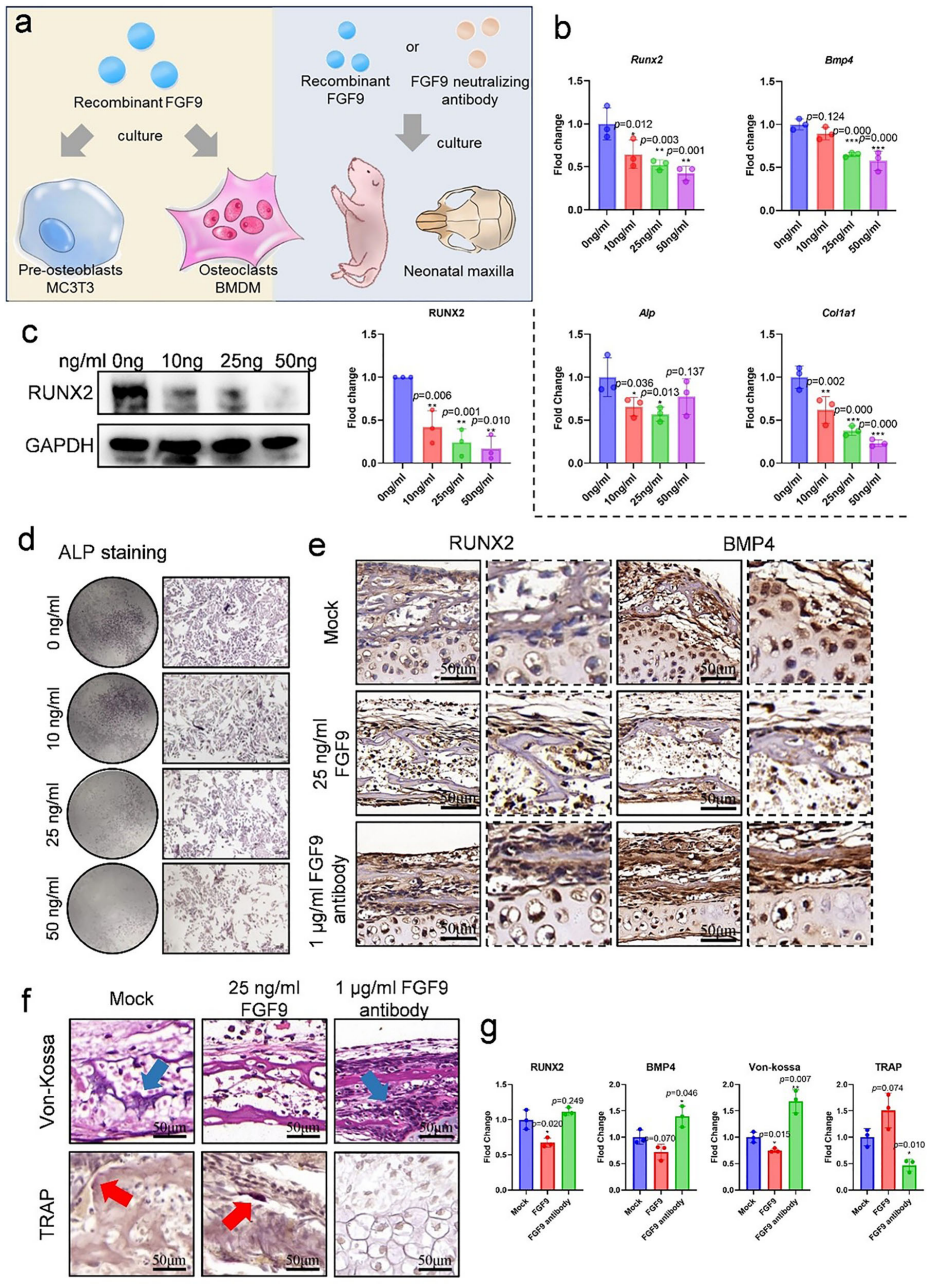
FGF9 inhibited osteogenesis and neonatal maxillary bone remodeling in vitro. a) Schematic diagram of experiments in this section. b–d) Osteogenic differentiation of MC3T3 after 0–50 ng /mL recombinant FGF9 protein stimulation: b) qPCR analysis of *Runx2, Bmp4, Oc*, and *Alp* mRNA expression; c) Representative immunoblot images and quantification of RUNX2 protein levels; d) Representative ALP staining images. e) Representative immunohistochemical images for RUNX2 and BMP4 of neonatal maxilla following treatment with 25 ng/mL recombinant FGF9 protein or 1 µl/mL FGF9 neutralizing antibody. The dashed boxes represent magnified images. f) Von Kossa and TRAP staining of neonatal maxilla following treatment with 25 ng/mL recombinant FGF9 or 1 µl/mL FGF9 neutralizing antibody. Blue arrows indicate the mineralized staining areas, while red arrows denote the TRAP staining regions. g) Quantification of RUNX2, BMP4, Von‐kossa and TRAP staining. Scale bar = 50 µm. Data are presented as mean ± SD. *n* = 3. Statistical comparison between groups was performed using an independent samples *t*‐test. Statistical significance was determined as follows: *p* < 0.05*, *p* < 0.01 **, *p* < 0.001***.

After determining the role of FGF9 from osteocytes under mechanical stress, we then investigated the direct effects of FGF9 by using recombinant protein on preosteoblasts and osteoclasts in vitro (**Figure** **3**a). With 10–50 ng/mL FGF9 stimulation for 24 h, there was an obvious reduction in osteogenesis on MC3T3, evidenced by lower levels of transcripts and proteins associated with osteogenic differentiation (*Runx2* and *Bmp4*) and mineralization markers (*Col1a1* and *Alp*) (Figure 3b,c). Consistent with these findings, ALP staining also revealed a concentration‐dependent inhibition of osteogenic mineralization (Figure 3d; Figure S8g, Supporting Information). Furthermore, treatment with 25 ng/mL FGF9 significantly promoted the fusion of bone marrow‐derived macrophages (BMDMs) and enhanced osteoclastogenesis (Figure S8a–e, Supporting Information). Despite its direct regulation, FGF9 also influenced the OPG/RANKL ratio in osteoblasts (Figure S8f, Supporting Information), suggesting an indirect regulation between osteoblasts and osteoclasts as well.

Subsequently, to validate these in vitro findings in an ex vivo model, neonatal maxilla explants were cultured with recombinant FGF9 protein and its neutralizing antibody (Figure 3a). Immunohistochemical analysis showed that treatment with 25 ng/mL FGF9 reduced the expression of RUNX2 and BMP4 compared to the Mock group, accompanied by diminished mineralization as shown by Von Kossa staining (Figure 3e–g). Additionally, osteoclastogenesis in the maxilla was enhanced, shown by TRAP staining (Figure 3f,g). In contrast, administration of the FGF9 neutralizing antibody resulted in increased levels of osteogenic markers, promoted mineralization, and inhibited osteoclast activity (Figure 3e–g). Collectively, these results identify FGF9 as a negative regulator of bone formation, which inhibited osteogenesis and promoted osteoclastogenesis.

### 2.4 FGF9 Induces Osteogenic Inhibition on Preosteoblasts via ATF5 and NR2F1

**Figure 4:**
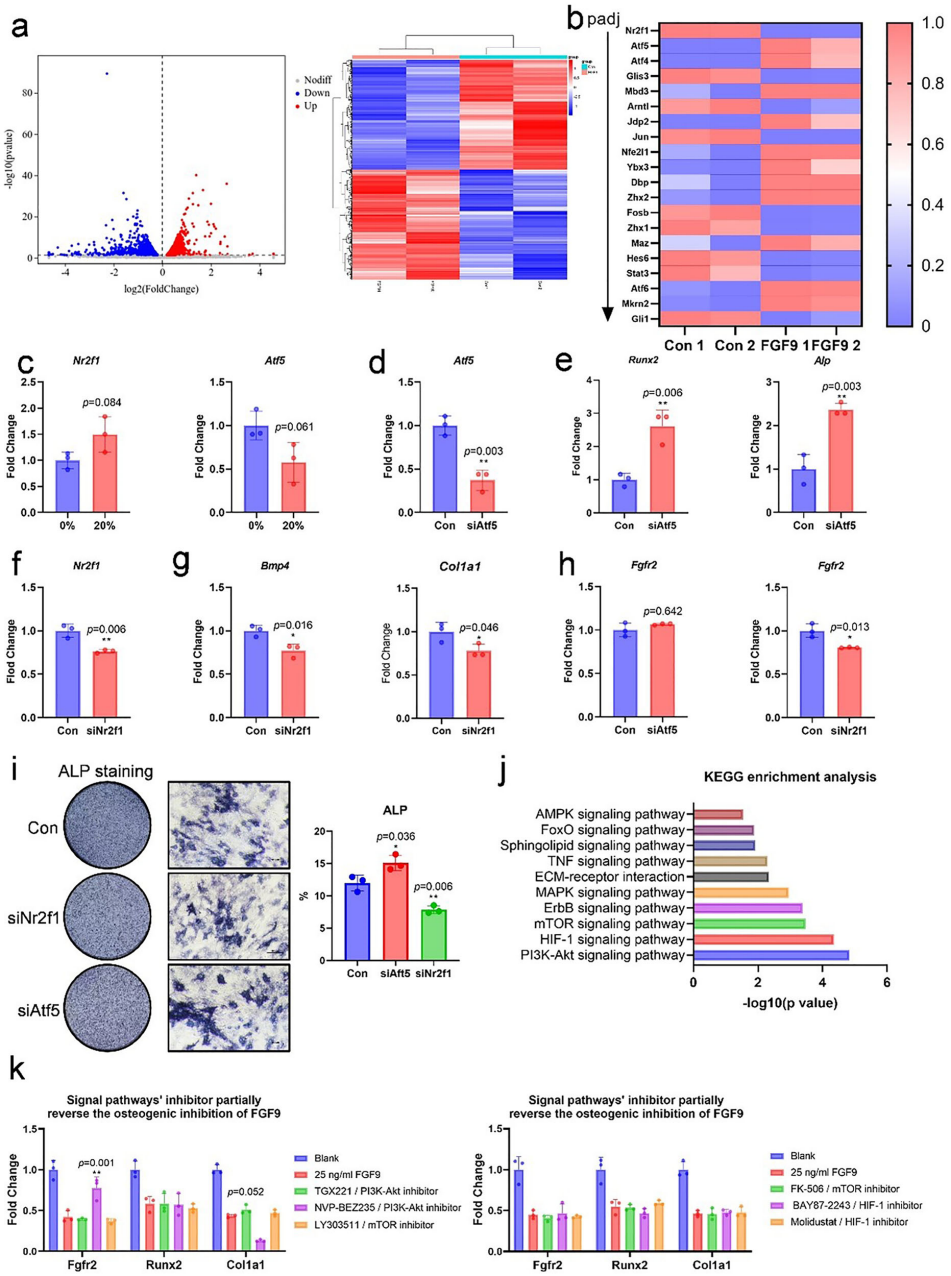
FGF9 negatively regulated osteogenesis through ATF5 and NR2F1 independent of PI3K‐Akt, HIF‐1, and mTOR signaling pathway. a,b,j) RNA sequencing from MC3T3 treated with 25 ng/mL FGF9 for 24 h: a) Volcano plot and clustering analysis. b) Heatmap of differential transcription factors. j) KEGG pathway analysis. c) mRNA expression of *Nr2f1* and *Atf5* in preosteoblasts cultured with conditioned medium from osteocytes subjected to 20% mechanical stress. d–i) Osteogenic differentiation of MC3T3 after siRNA‐mediated knockdown of *Nr2f1* and *Atf5*: d,f) mRNA levels of *Nr2f1* and *Atf5*. e,g) qPCR analysis of *Runx2, Alp, Bmp4* and *Col1a1* mRNA expression. h) mRNA levels of *Fgfr2*. i) Representative ALP staining images and its quantification. k) mRNA expression of osteogenic markers *Runx2, Col1a1* and *Fgfr2* in MC3T3 following treatment with FGF9 and corresponding signaling pathway inhibitors of PI3K‐Akt, HIF ‐ 1 and mTOR. Scale bar = 100 µm. Statistical comparison between groups was performed using an independent samples t‐test. Data are presented as mean ± SD. *n* = 3. Statistical significance was determined as follows: *p* < 0.05*, *p* < 0.01 **, *p* < 0.001***.

To evaluate the biological function of FGF9 on preosteoblasts, MC3T3 were treated with 25 ng/mL FGF9 and subjected to RNA sequencing to investigate the underlying mechanisms. Differential expression analysis revealed 64 significantly upregulated genes and 335 significantly downregulated genes (*p* < 0.05, |logFC| > 1; **Figure** **4**a), including several transcription factors. We then screened the differentially expressed transcription factors (Figure 4b) and selected the most significantly upregulated factor (*Atf5*) and the most significantly downregulated factor (*Nr2f1*) to further elucidate the mechanism.

First, we confirmed the expression trends of *Atf5* and *Nr2f1* in preosteoblasts cultured with conditional medium from osteocytes subjected to 20% mechanical stress. The expression of *Atf5* and *Nr2f1* were correspondingly decreased and increased, indicating that these factors may mediate the effects of osteocyte‐derived FGF9 on osteogenesis (Figure 4c). To clarify their functional roles in osteogenic differentiation, we then employed siRNAs targeting *Atf5* and *Nr2f1* (Figure 4d,f). Our results revealed that ATF5 negatively regulated osteogenic differentiation, evidenced by increased expression of *Runx2* and *Alp* (Figure 4e,i). In contrast, the silence of *Nr2f1* in preosteoblasts decreased the levels of *Bmp4* and *Col1a1*, and reduced ALP activity (Figure 4g,i). Overall, our findings suggest that FGF9 inhibits osteogenesis in preosteoblasts via modulation of ATF5 and NR2F1.

After FGF9 stimulation, signaling pathways are required to transmit the signal from the cell membrane to the nucleus, regulating transcription factor expression and influencing downstream osteogenic gene transcription. To identify the specific pathways involved, we performed KEGG analysis, which revealed that FGF9 activated several signaling cascades, with the PI3K‐Akt, HIF‐1, and mTOR pathways being the most prominent (Figure 4j). To determine whether these pathways mediate the osteogenic inhibition induced by FGF9, MC3T3 were cultured with FGF9 and correspondent inhibitors for 24 h. The PI3K‐Akt inhibitors (TGX221 and NVP‐BEZ235) moderately reversed FGF9's inhibitory effects on *Col1a1* and *Fgfr2* expression, whereas inhibitors targeting HIF‐1 (BAY87‐2243 and Molidustat) and mTOR (LY303511 and FK‐506) exhibited no significant effect compared to the Sham group (Figure 4k). Collectively, these results suggest that PI3K‐Akt, HIF‐1, and mTOR signaling pathways play limited roles in FGF9‐induced osteogenic inhibition. There might be other, yet‐to‐be‐identified mechanisms, responsible for transmitting the signal from the cell membrane to the nucleus following FGF9 stimulation, thus regulating the levels of ATF5 and NR2F1.

### 2.5 FGF9 Bound with FGFR2 and Induced its Nuclear Translocation in Preosteoblasts

**Figure 5:**
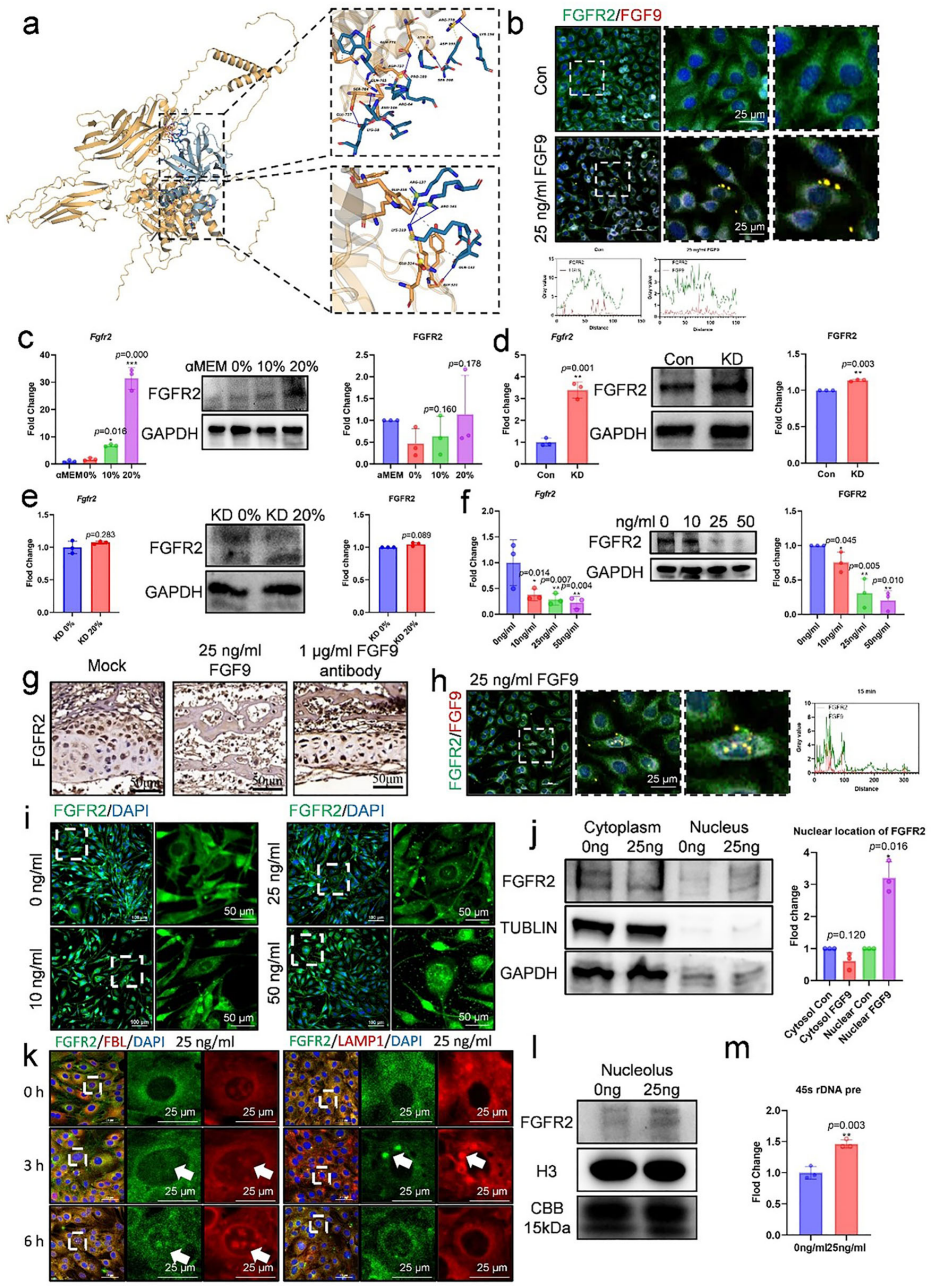
FGF9 interacted with FGFR2, inducing its nuclear translocation and resulting in an overall decrease in FGFR2 level. a) AlphaFold2‐based structural prediction of the binding interface between mouse FGF9 and FGFR2. b) Dual immunofluorescence staining and quantification of MC3T3 treated with 25 ng/mL recombinant FGF9 protein for 5 min. Dashed boxes indicate magnified images. Scale bar = 25 µm. c–f) mRNA and protein expression of FGFR2 in MC3T3 following co‐culture with osteocytes or treatment with recombinant FGF9: c) co‐culturing with osteocytes’ mechanical stress conditional medium. d) co‐culturing with osteocytes’ FGF9 knockdown conditional medium. e) co‐culturing with osteocytes’ FGF9 knockdown and mechanical stress conditional medium. f) culturing with 0–50 ng/mL recombinant FGF9 protein. g) Representative immunohistochemical images of FGFR2 in neonatal mouse maxilla following treatment with 25 ng/mL FGF9 recombinant protein or its 1 µl/mL neutralizing antibody. Scale bar = 50 µm. h) Dual immunofluorescence staining of MC3T3 treated with 25 ng/mL recombinant FGF9 protein for 15 min. Scale bar = 25 µm. i) Representative immunofluorescence images of FGFR2 in MC3T3 after 24 h of 25 ng/mL FGF9 treatment. Dashed boxes indicate magnified images. Scale bar = 50 µm. j) Western blot analysis and quantification of cytoplasmic and nuclear FGFR2 in MC3T3 after 48 h of 25 ng/mL FGF9 treatment. k) Dual immunofluorescence staining of FGFR2 with FBL or LAMP1 in MC3T3 after 3 and 6 h of 25 ng/mL FGF9 treatment. White arrows indicate the regions of co‐localization. Scale bar = 25 µm. l) Western blot analysis of nucleolar FGFR2 in MC3T3 after 48 h of 25 ng/mL FGF9 treatment. m) mRNA expression of the 45s rDNA precursor in MC3T3 following 25 ng/mL FGF9 treatment for 24 h. Data are presented as mean ± SD. *n* = 3. Statistical comparison between groups was performed using an independent samples *t*‐test. Statistical significance was determined as follows: *p* < 0.05*, *p* < 0.01 **, *p* < 0.001***.

As the receptor with the highest affinity for FGF9,^[^ ^24^ ^]^ FGFR2 is expressed during maxillary development, and functions as a positive regulator of osteogenesis (Figure S5a,b,d,e, Supporting Information). We investigated their binding on preosteoblasts using computational predictions and dual immunofluorescence staining. AlphaFold2 confirmed the high affinity between FGF9 and FGFR2 by predicting specific amino acid binding sites, and phosphorylation sites were detected by mass spectrometry (**Figure** **5**a; Figure S11a,b, Supporting Information). In vitro, the FGF9–FGFR2 complex was observed on the membrane of MC3T3 as early as 5 min after treatment with 25 ng/mL FGF9 (Figure 5b).

We next examined FGFR2 expression following FGF9 stimulation. Our results indicate that the mRNA and protein level of FGFR2 showed a notable increase with the conditional medium of osteocytes with mechanical stress or FGF9 knockdown (Figure 5c,d), whereas no significant change was observed under KD 20% CM conditions (Figure 5e). Furthermore, treatment with recombinant FGF9 (10–50 ng/mL) resulted in a dose‐dependent suppression of FGFR2 expression (Figure 5f). A corresponding trend was observed in neonatal maxilla tissues treated with FGF9 or its neutralizing antibody (Figure 5g; Figure S7n, Supporting Information). The function of FGFR2 was also confirmed by a Lenti‐virus overexpression experiment (Figure S11c, Supporting Information). Overexpression of FGFR2 significantly enhanced the osteogenesis by increased expression of osteogenic and mineralization markers, and enhanced ALP staining (Figure S11d,e, Supporting Information). Therefore, these results demonstrate that FGF9 inhibits FGFR2 expression in preosteoblasts.

Next, to elucidate the fate of FGFR2 in preosteoblasts, we unexpectedly observed that after 15 min by dual immunofluorescence experiments, a portion of the FGF9–FGFR2 complexes had translocated into the nucleus (Figure 5h), suggesting that FGF9 might induce the nuclear translocation of FGFR2. To further investigate this phenomenon, we treated MC3T3 with 0–50 ng/mL FGF9 and analyzed the subcellular distribution of FGFR2. With the stimulation of FGF9 for 24 h, a significant amount of FGFR2 was detected in the nucleus (Figure 5i; Figure S11f, Supporting Information). The location of nuclear FGFR2 was further demonstrated by immunoblotting, showing decreased FGFR2 expression in the cytoplasm and increased expression in the nucleus (Figure 5j). Moreover, the extent of nuclear localization of FGFR2 was positively correlated with the FGF9 concentration. (Figure 5i).

However, with 3 h of stimulation with 25 ng/mL FGF9, FGFR2 clearly colocalized with the lysosomal marker LAMP1, though this signal disappeared by 6 h (Figure 5k; Figure S11g, Supporting Information). In contrast, within the nucleus, colocalization of FGFR2 with the nucleolar marker FBL was evident at 3 h and became more pronounced by 6 h. The presence of FGFR2 in the nucleolus was further confirmed by immunoblotting (Figure 5l). Furthermore, the nucleolus is involved in the synthesis and processing of rRNA. Our results showed that FGF9 regulates the transcription of 45s rDNA, indicating the effect of FGFR2 on the nucleolus (Figure 5m).

Altogether, these results suggested that after combining with FGF9, a portion of FGFR2 on the membrane is degraded in lysosomes, while the remaining FGFR2 translocated to the nucleolus and induced sustained nuclear signal, leading to the inhibition of osteogenesis.

### 2.6 Overexpression of FGF9 Contributes to the Occurrence of Underdeveloped Maxilla

**Figure 6:**
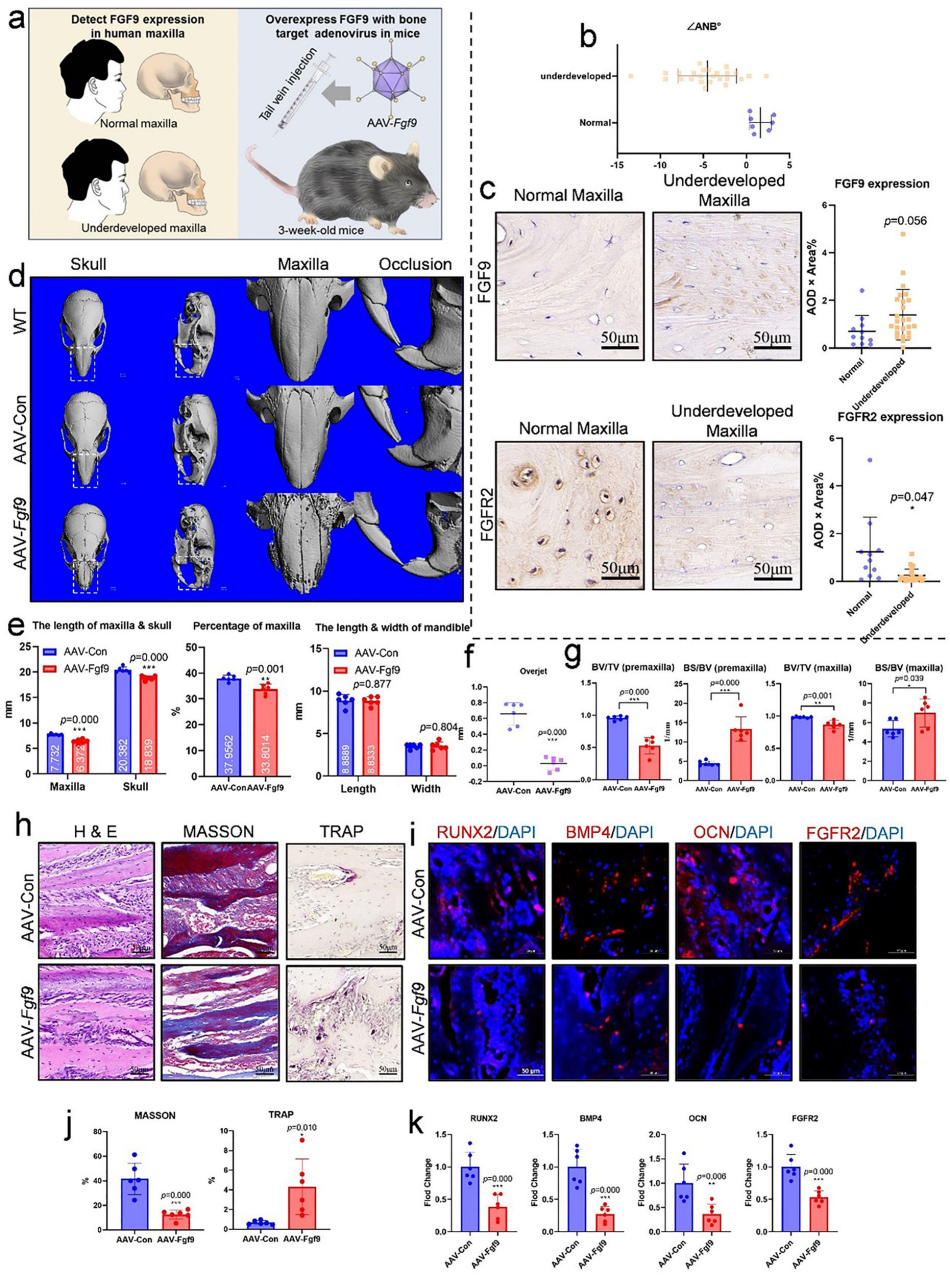
Overexpression of FGF9 suppressed maxillary development through the inhibition of osteogenesis and activation of osteoclastogenesis. a) Schematic diagram of experiments in this section. b) The ANB angles in patients with normal maxilla versus those with maxillary underdevelopment. c) Representative immunohistochemical images and quantification of FGF9 and FGFR2 expression in normal maxilla and underdeveloped maxilla. d–h) Overexpressing FGF9 through bone‐targeted adenovirus (*n* = 6): d) Representative 3D Micro‐CT reconstruction images of skulls, with magnified views of the maxillary region and the incisor area. e,f) The quantification of maxillary length, mandible width and length, skull length, and the incisors’ overjet. g) Quantification of BV/TV and BS/BV in the premaxilla and maxilla. h,j) Representative images and quantification of H&E, Masson, and TRAP staining of the maxilla. i,k) Representative immunofluorescence images and quantification of RUNX2, BMP4, OCN, and FGFR2 expression in the maxilla. Scale bar = 50 µm. Data are presented as mean ± SD. Statistical comparison between groups was performed using an independent samples *t*‐test. Statistical significance was determined as follows: *p* < 0.05*, *p* < 0.01 **, *p* < 0.001***.

Considering the role of FGF9 in sensing mechanical stress, we thought it may contribute to the pathogenesis of maxillary underdevelopment. To investigate this possibility, we performed immunohistochemical analysis on 11 normal maxilla cases and 25 cases of underdeveloped maxilla from human (**Figures** **6**a and S10, Supporting Information). The results revealed that the immunohistochemical intensity of FGF9 was elevated in the underdeveloped group (Figure 6b,c), indicating that FGF9 might be a potential pathogenic factor. In contrast, FGFR2 expression was notably decreased in the underdeveloped group (Figure 6c), supporting the notion that the FGF9–FGFR2 signaling axis between osteocytes and preosteoblasts plays a critical role in regulating maxillary development.

Next, we engineered a bone‐targeted adenovirus (AAV) to overexpress FGF9 in vivo (Figure 6a). The AAV‐*Fgf*9 mice showed lower weight at 2‐month age (Figure S10b, Supporting Information). After confirming the transfection efficiency and biosecurity of the AAV‐*Fgf9* system (Figure S9b–e, Supporting Information), we evaluated the maxillary development via Micro‐CT. Compared to the control group (AAV‐Con), the FGF9 overexpression group (AAV‐*Fgf9*) exhibited significant maxillary bone destruction (Figure 6d,g), and a markedly underdeveloped maxilla with shortened maxillary length (Figure 6e). Interestingly, mandibular length and width remained unchanged (Figure 6e), resulting in an edge‐to‐edge or even reverse occlusion (Figure 6f). H&E staining revealed a loose suture in the maxilla of AAV‐ *Fgf9* group (Figure 6h). Overexpression of FGF9 inhibited bone formation, as demonstrated by reduced expression of osteogenic markers RUNX2, BMP4, and OCN, along with diminished mature bone structures observed via Masson staining (Figure 6h–k). Meanwhile, osteoclast activity was significantly increased, as evidenced by enhanced TRAP staining in the maxilla (Figure 6h,j). Consistent with the impaired development and bone destruction of the maxillary bone, the femur in the overexpression group also showed length reduction, cortical bone thickness reduction, cancellous bone destruction, reduced osteogenesis, and enhanced osteoclasts (Figure S10a,c–g, Supporting Information). In addition, FGFR2 levels were decreased in AAV‐*Fgf9* group (Figure 6i). Therefore, these findings suggest that FGF9 overexpression suppressed osteogenesis, promoted osteoclastogenesis, and contributes to the pathology of maxillary underdevelopment.

## 3 Discussion

**Figure 7:**
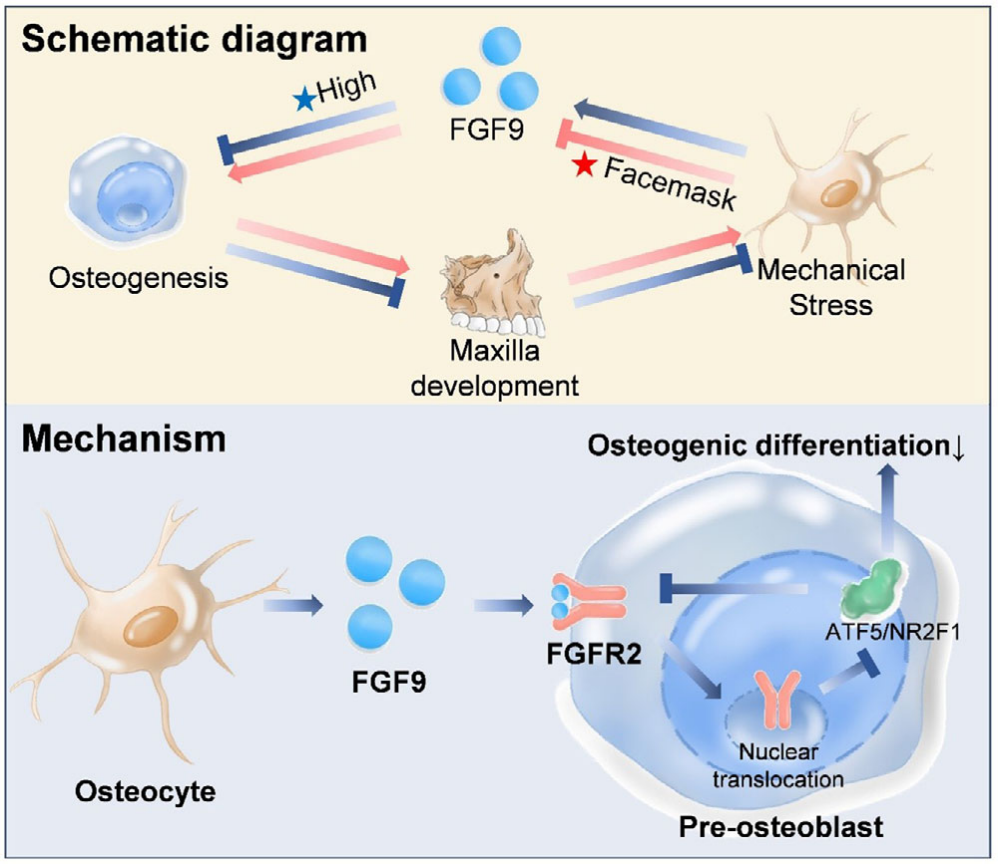
Schematic diagram of the study. Abnormally high FGF9 expression inhibits osteogenic differentiation, leading to maxillary underdevelopment. Insufficient mechanical signaling in the underdeveloped maxilla further drives FGF9 overexpression. Conversely, supplementing mechanical signals reduces FGF9 secretion, thereby promoting osteogenic differentiation and facilitating maxillary development. FGF9, secreted by osteocytes, binds to FGFR2 receptors on preosteoblasts, triggering the nuclear translocation of FGFR2. Nuclear FGFR2 modulates the transcription factors ATF5 and NR2F1, which in turn downregulate FGFR2 protein expression and activate downstream signals that inhibit osteogenesis.

In our study, we demonstrated for the first time that mechanical stress significantly downregulates FGF9 expression in osteocytes, thereby modulating the activity of preosteoblasts. Inhibition of FGF9 can enhance osteogenic differentiation and mineralization while suppressing osteoclast differentiation, ultimately promoting maxillary development. Mechanistically, we found that FGF9 can bind to FGFR2 on the plasma membrane of preosteoblasts, which induces the nuclear translocation of FGFR2 to the nucleolus. This translocation subsequently alters the expression of key transcription factors, including ATF5 and NR2F1, leading to the inhibition of osteogenic differentiation (**Figure** **7**).

The previous studies have primarily focused on osteocyte‐secreted factors such as sclerostin.^[^ ^25^ ^]^ Importantly, we identified fibroblast growth factor 9 (FGF9) was another important bone‐specific paracrine factor abundantly secreted by osteocytes. FGF9 functions as a negative regulator of osteogenesis and exerts complex effects.^[^ ^18^, ^19^, ^26^, ^27^ ^]^ FGF9 knockout mice exhibit impaired bone formation and reduced bone mass.^[^ ^18^, ^28^, ^29^, ^30^ ^]^ FGF9 S99N (loss‐of‐function) mutation leads to excessive osteogenesis and joint fusion.^[^ ^19^ ^]^ Whereas FGF9 overexpression causes delayed osteogenesis and abnormal chondrogenesis.^[^ ^28^ ^]^ In this study, FGF9 expression was significantly downregulated in osteocytes subjected to mechanical loading, suggesting that mechanical forces inhibit FGF9‐mediated signaling to favor bone formation. The alterations of mechanical signal distribution within the maxillary tuberosity can impair the osteocyte‐driven FGF9 paracrine network, which further disrupts maxillary growth. FGF9 significantly suppresses osteogenic differentiation and promotes osteoclasteogenesis in the development of maxilla. Here, we propose a two‐stage loop: high expression of FGF9 initiates maxillary deficiency, which in turn leads to insufficient mechanical unloading, exacerbating FGF9 upregulation and hindering maxillary growth. Encouragingly, mechanical stimulation can interrupt this cycle and promote osteogenic activity, offering a potential therapeutic strategy for maxillary underdevelopment.

In the mechanosensitive process, we demonstrated the important role of FGF9, and FGF9 may have crosstalk with other classical mechanosensitive pathways. Several studies have shown that Wnt signaling can regulate FGF9 expression in different cell types.^[^ ^29^, ^30^, ^31^ ^]^ Given the pivotal role of Wnt signaling in osteocyte mechanotransduction, it is plausible that FGF9 is modulated by Wnt‐related transcriptional regulation. However, the precise mechanisms remain to be clarified in future studies.

Interestingly, the mechanism by which FGF9 inhibits osteogenesis appears to diverge from the classical signaling paradigm of fibroblast growth factor receptors. FGFs typically activate receptor‐mediated pathways such as MAPK, PI3K/Akt, and PLCγ through FGFR2 at the plasma membrane.^[^ ^32^, ^33^, ^34^, ^35^ ^]^ Although our result revealed upregulation of the PI3K/Akt pathway, pharmacological inhibition of these canonical downstream cascades failed to reverse the FGF9‐induced suppression of osteogenic differentiation.^[^ ^19^ ^]^ FGF9 treatment can trigger a striking translocation of FGFR2 from the plasma membrane to the nucleus, with pronounced accumulation in the nucleolus. FGF9 may facilitate FGFR2 nuclear entry by functioning as a carrier, as it possesses a putative NLS itself (Figure S11h, Supporting Information). Inhibition of the conventional lysosomal degradation pathway further enhances FGF9‐induced nuclear localization of FGFR2, thereby amplifying its suppressive effect on osteogenesis (Figure S11i,j, Supporting Information). This nuclear trafficking of FGFR2 is a novel finding in the bone and represents a critical mechanistic shift. Furthermore, nuclear FGFR2 may modulate the expression of transcription factors ATF5 and NR2F1. Specifically, NR2F1 has been shown to promote osteogenesis,^[^ ^36^ ^]^ whereas ATF5 acts as an inhibitory factor.^[^ ^37^ ^]^ The nuclear‐translocated FGFR2, driven by FGF9 can suppress the transcriptional factors' expression.

In conclusion, our study uncovers a novel mechanistic basis for targeting maxillary underdevelopment, highlighting the molecular therapeutic potential of combining mechanical stimulation with FGF9 modulation. These findings also expand the current understanding of osteocyte–preosteoblast communication and crosstalk. However, several limitations must be addressed before clinical application can be realized. First, the facemask protraction model utilized WT mice due to the lack of a genetically model for maxillary underdevelopment. Second, as a multifactorial disease, maxillary underdevelopment involves complex pathogenic factors beyond the FGF9‐FGFR2 axis, necessitating further investigation. Future research should focus on refining disease models and optimizing therapeutic strategies for maxillary underdevelopment.

## 4 Experimental Section

### Ethics Statement

All relevant ethical regulations for animal and human research have been complied with. All experimental animal procedures were approved by the Animal Experiment Ethics Committee of Nanjing University (approval number: IACUC‐D2303101). All experiments involving human samples and clinical data were approved by the Ethics Committee of Nanjing Stomatological Hospital (approval number: NJSH‐2023NL‐10) and written informed consent was obtained from all participants prior to enrollment.

### 3D Fine Element Analysis (FEA)

CBCT data (NEWTOM VGi, Italy) of normal and undeveloped maxilla were acquired from a skeletal class III patient with undeveloped maxilla and underwent orthognathic surgery. First, the data were imported into MIMICS 20.0 software (Materialize, Leuven, Belgium) to generate 3D base models. Then, GEOMAGIC Studio 2014 (Rain‐dropGEOMAGIC, North Carolina, USA) was used to optimize the basic model and create a surface model structure. With the help of NX1911 software (Siemens, German), the teeth, the periodontal ligaments, and jaws were assembled and the 3D CAD (computer‐aided design) models were obtained. Finally, models were imported into ANSYS Workbench 2019 (ANSYS, Pennsylvania, USA) to generate a 3D finite element model for FEA (Figure S1a and Table S1, Supporting Information). Then, masticatory muscle strength was added to normal maxillary and underdeveloped maxillary models according to literature^[^ ^38^ ^]^ (Figure S1b and Table S2, Supporting Information), to analyze the force on the maxillary tubercle.

### GEO RNA‐Seq Data Analysis

RNA‐seq data from GEO database were used to analyze the expression of *Fgf9* during embryonic period (GSE161126) and expression of *Fgf9* under flow shear stress (GSE144265). Data were uploaded and analyzed on NetworkAnalyst website to obtain volcano map and protein interaction map.^[^ ^39^ ^]^

### Human Maxilla Samples

Maxillary bone samples were collected from 11 patients with normal maxilla and 25 patients with underdeveloped maxilla to analyze the expression levels of FGF9 and FGFR2. Baseline demographic and clinical information of all patients is presented in Table S10 (Supporting Information). For patients with available lateral cephalograms, the ANB angle was measured to assess skeletal relationships (Figure 6b).

### Human Cephalometric Analysis

Lateral cephalometric radiographs were collected from adolescent patients before and after treatment (with or without facemask protraction). Cephalometric measurements were performed using Dolphin Imaging software, and the sagittal length of the maxilla was assessed using the distance from ANS (anterior nasal spine) to PNS (posterior nasal spine) as the primary parameter. A total of 20 patients with normal maxilla without facemask protraction and 18 patients with underdeveloped maxilla underwent facemask protraction were included in the cephalometric analysis (Table S9, Supporting Information).

### Mice

All C57B/L6J mice were purchased from the Jiangsu Huachuang Sino Pharmaceutical Technology Co., Ltd. The care and handling of the experimental animals were conducted in accordance with the requirements of the Animal Experiment Ethics Committee of Nanjing University under specific pathogen‐free (SPF) conditions.

### Mice Treated with Facemask Traction

Six‐week‐old male C54B/L6J mice were randomly divided into two groups. Sham group: No anterior maxillary traction was performed, only a dorsal brace was created. Facemask group (FM group): Maxillary anterior traction modeling was conducted with 20 g force (Figure S2d and Table S3, Supporting Information). The modeling steps are shown in Figure S2c (Supporting Information) and the force of 20 g was measured by a miniature flat‐type force gauge. For each experiment, six mice were consisted in each group with modeling durations of 1 or 2 weeks. After modeling, the mice were anesthetized and euthanized through cervical dislocation for sample collection.

### Mice Treated with AAV‐Fgf9 Tail Suspension

The bone‐targeted *Fgf9* (Mouse, NM_013518.4) overexpression adenovirus was constructed by Shanghai Heyuan Biotechnology Co., Ltd. The virus composition is as follows: pAAV‐CMV‐EF1‐GdGreen‐WPRE (AAV‐Con); pAAV‐CMV‐Fgf9‐3xFlAG‐EF1‐GdGreen‐WPRE (AAV‐*Fgf9*) (Figure S9a, Supporting Information). Twelve 3‐week‐old male C57B/L6J mice were randomly divided into two groups. Each mouse received a tail vein injection of AAV‐Con or AAV‐*Fgf9* virus at a dosage of 8.0E+11. After the injection, the mice were housed for 5 weeks. At 2 months of age, the mice were anesthetized and euthanized via cervical dislocation for sample collection.

### Micro‐CT Analysis

Mice skull and femur samples were harvested, soft tissues were removed, and the remaining tissues were stored in 4% paraformaldehyde (PFA) overnight. Then, skull and femur samples were scanned with a SCANO Medical AG machine at an X‐ray voltage of 57 kV, a current of 184 µA, and a resolution of 10 µm per pixel. Femur width, cortical bone thickness, bone volume fraction (BV/TV), bone mineral density (BMD), surface‐to‐volume ratio (BS/BV), trabecular number (Tb.N), trabecular separation (Tb.Sp), and trabecular thickness (Tb.Th) were measured in the machine (Figure S9b, Supporting Information). The lengths of the maxilla, skull, and femur were measured by Image J 1.8 after 3D reconstruction.

### Immunohistochemical Staining and Immunofluorescence

Bone tissues were fixed in 4% PFA for 48 h and incubated in 10% EDTA for decalcification. Then, specimens were embedded in paraffin and sectioned at 5 µm. For immunohistochemical staining, sections were dewaxed and rehydrated. A solution of 3% H_2_O_2_ was used to block the activity of endogenous peroxidase. Antigen retrieval was performed with pepsin (Sangon Biotech, E673007) at 37 °C for 30 min. The sections were then blocked with goat serum. The primary antibody was added and incubated overnight at 4 °C. Anti‐rabbit secondary antibody conjugated with HRP was added and incubated for 40 min at room temperature, followed by color development with a DAB kit (Maxin Biotechenologies, DAB‐2031). For immunofluorescence, sections were blocked and stained with primary antibody overnight in the same way. Anti‐rabbit secondary antibodies with fluorescein were used as the secondary antibody and sections were incubated for 2 h at room temperature to avoid the light. DAPI (Beyotime Biotechnology, C1005) was used for nuclear staining. The antibodies used are shown in Table S4 (Supporting Information).

### Analysis of Bone Phenotypes

For EdU staining, EdU (Beyotime Biotechnology, C0071S) was prepared using PBS at a dosage of 50 mg kg^−1^ and administered via intraperitoneal injection 4 h prior to euthanizing the mice. After 4 h, samples were collected and sectioned. After dewaxing, 50 µL of Click reaction solution was added to each slide and incubated in the dark at room temperature for 30 min for EdU visualization. For TUNEL staining (Vazyme Biotechnology, A112‐01), after dewaxing, sections were permeabilized with Proteinase K solution. After equilibration at room temperature, the sections were incubated in the dark with TdT labeling solution at 37 °C for 1 h. DAPI was used for nuclear staining. For histological analysis, sections were stained by H&E, MASSON (Solarbio Life Sciences, G1346), ALP (Beyotime Biotechnology, C3206), Von‐Kossa (Wordan, Shanghai, China), TRAP (Solarbio Life Sciences, G1492) staining kit according to the standard protocol after de‐waxing. After staining, the sections were dehydrated and cleared with alcohol and xylene, and finally mounted with neutral resin.

For histological analysis, all samples were harvested from the posterior maxilla of mice, corresponding anatomically to the maxillary tuberosity region in humans. Quantification of the mature bone area (stained red in MASSON), osteoclastic area (stained wine red in TRAP), osteogenic area (stained blue in ALP) and mineralized area (stained black in Von‐kossa) was performed using ImageJ software to get the positive area and calculate the average value and the differences between groups.

### Tyramide Signal Amplification (TSA) Staining

After treatment with H_2_O_2_, deparaffinization, antigen retrieval, and blocking, the sections were incubated with the first primary antibody overnight at 4 °C in the dark. The sections were then incubated with an HRP‐conjugated anti‐rabbit secondary antibody for 50 min, followed by incubation with 50 µL of TSA‐488 (ABclonal, RK05902) staining solution for 10 min at room temperature in the dark to complete the labeling of the first primary antibody. Next, the sections were treated with an antibody elution solution (ABclonal, RM02984) at 37 °C for 20 min and repeated once to remove nonspecific binding. Finally, the second primary antibody was incubated, and after binding with the HRP secondary antibody, TSA‐555 (ABclonal, RK05902) staining solution was applied for labeling. DAPI was used for nuclear staining.

### Cell Culture

The osteocyte used was the MLO‐Y4 cell line (a gift from Pro. Baosheng Guo's lab) and was cultured in α‐MEM medium supplemented with 5% fetal bovine serum (FBS), 5% calf serum, and 1% penicillin‐streptomycin (Osteocyte culture medium). The medium was refreshed every 24 h, and cells were passaged upon reaching 80% confluence. For osteoblasts, the MC3T3 cell line (Cyagen Biosciences, Suzhou, China) was cultured in α‐MEM medium containing 10% FBS and 1% penicillin‐streptomycin (α‐MEM complete medium). Medium was changed every 48 h, and cells were passaged when confluence reached 80%. MC3T3 were treated with recombinant mouse FGF9 protein (Cusabio, CSB‐AP004131MO) at concentrations ranging from 0 to 50 ng/mL, and/or 10 µm chloroquine (Aladdin, C193834) for the indicated durations to perform further experiments. Primary periosteal stem cells (PSCs) were isolated from the cranial sutures of neonatal mice. Bilateral 1 mm strips of cranial suture tissue were collected, minced, and digested in α‐MEM medium containing 0.2% type II collagenase for 1 h at 37 °C with shaking. The digested cells were filtered through a 70 µm mesh and plated after centrifugation. PSCs were cultured in α‐MEM complete medium, with half‐medium changed every 2 d initially, and then complete medium changed every 48 h. Passaging was performed at 80% confluence. For osteogenic induction of MC3T3 cells and PSCs, α‐MEM complete medium was supplemented with osteogenic inducers (50 mg L^−1^ vitamin C, 0.5 mmol L^−1^ β‐glycerophosphate, and 10^−8^ mol L^−1^ dexamethasone) to create the osteogenic induction medium, with medium changes every 72 h. After 7 days of induction, ALP staining was conducted. Quantification of ALP staining was performed using ImageJ by selecting and measuring the area of dark blue positively stained regions.

Primary bone marrow‐derived monocytes (BMDMs) were isolated from the long bones of 3‐week‐old WT mice. After red blood cell lysis (Beyotime Biotechnology, C3702) and filtration through a 70 µm mesh, the collected cells were cultured overnight in α‐MEM complete medium. Then, the supernatant was removed, and nonadherent cells were collected via centrifugation and seeded into new culture dishes. For osteoclast induction, cells were cultured in DMEM medium containing 10% FBS, 1% penicillin‐streptomycin, 30 ng/mL M‐CSF and 50 ng/mL RANKL (Osteoclast induction medium) with half‐medium changed every 3 d. After 7 days, experiments such as TRAP staining were performed to assess osteoclast differentiation level.

### Flexcell Treatment and Conditional Medium

Osteocytes were seeded onto Flexcell tension plates (BioFLEX, BF‐3001C) at 50% confluence. After overnight attachment, the cells were subjected to varying degrees of tensile strain (10% or 20%, 0.5 Hz) using the FX‐5000T system (Flexcell) for 12 or 24 h. The nonstressed control group (0%) was cultured in standard six‐well plates at the same time. The osteocyte medium was collected every 12 h. After the stretch force treatment, the culture supernatants of osteocytes were collected, centrifuged, and filtered through a 0.22 µm filter (Osteocyte supernatants). The concentration of FGF9 in the medium collected from control osteocytes after 12 h of culture was measured by ELISA and determined to be ≈0.603 ng/mL. The filtered supernatants were then mixed 1:1 with the corresponding culture medium of MC3T3 or BMDMs to obtain the osteocyte conditional medium (CM).

### Small Interfering RNA (siRNA) and Lentivirus Transfection

Both siRNA and lentivirus were constructed by Syngenbio Co., Beijing, China. For siRNA transfection, cells were seeded at a density of 0.5–2 × 10^5^ per well. After overnight incubation, when the cells reached ≈50% confluence, transfection was performed. Lipofectamine 2000 (Thermofisher, America) was used to encapsulate 50 nm siRNA, and the medium was changed 6 h after transfection. Subsequent experiments were performed 48 h later. The siRNA sequences are shown in Table S5 (Supporting Information). For the overexpression lentivirus of *Fgfr2* (Mouse, NM_010207), a multiplicity of infection (MOI) of 50 was chosen as the transfection condition. Cells were seeded at a density of 1 × 10^5^ per well in a six‐well plate. After overnight incubation, the medium was replaced with serum‐free medium, and lentivirus along with 5 µg/mL Polybrene (Beyotime Biotechnology, C0351), was added. The medium was changed to complete culture medium after 8 h of transfection, and subsequent experiments were performed 48 h later.

### In Vitro Maxilla Culture

After isolating from neonatal mice, the maxilla was cultured in α‐MEM complete medium. Within the corresponding groups, either 25 ng/mL recombinant FGF9 protein (Cusabio, CSB‐AP004131MO) or 1 µg/mL FGF9 neutralizing antibody (Bioss, bs‐5906R) was added. After culturing for 48 h, the samples were fixed with 4% PFA for subsequent tissue embedding and sectioning.

### Real‐Time Reverse Transcription PCR (RT‐PCR) Analysis

Total RNA was extracted using the RNA extraction Kit (Beyotime Biotechnology, R0026) or TRIzol (Sigma, T9424) and was reverse‐transcribed into cDNA using the reverse transcription kit (Vazyme, R212‐01). RT‐PCR was performed using the SYBR qPCR kit (Vazyme, Q311‐02) in Bio‐Rad 384 system. The primer sets used were as shown in Table S6 (Supporting Information).

### RNA‐Seq

MC3T3 were stimulated with 25 ng/mL of recombinant FGF9 protein for 48 h. Total RNA of MC3T3 was isolated with the RNA extraction Kit (Beyotime Biotechnology, R0026). Complementary DNA library preparation and sequencing were performed by Shanghai Peisenor Biotechnology Co., LTD. Per kilo base per million (FPKM) was used as an expression value for each gene and the analysis was performed on the company's website (https://www.genescloud.cn/).

### Mass Spectrometry

MC3T3 were stimulated with 25 ng/mL of recombinant FGF9 protein for 15 min. Total protein was extracted using a lysis buffer containing phosphatase inhibitors (Beyotime Biotechnology, P0013, P0013B, P1045). Mass spectrometry analysis was conducted by Shanghai Peisenor Biotechnology Co., LTD., employing a shotgun approach to examine amino acid modifications. Following peptide digestion, chromatographic separation, and mass spectrometry identification of the protein samples, phosphorylation sites on FGFR2 were selected.

### Western Blotting

For western blot analysis, total protein of cells was obtained with Protein Lysis Buffer (Beyotime Biotechnology, P0013, P0013B, P1045) and quantified by a BCA kit (Beyotime, P0011). Subsequently, the proteins were denatured with SDS buffer, followed by separation using 4–20% or 8% SDS‐PAGE and membrane transfer according to standard protocols. After blocking with a rapid blocking solution (Sevren Bio, G2052), the membrane was cut according to the molecular weight of the target protein and incubated with the primary antibody overnight at 4 °C. Following this, the membrane was incubated with an HRP‐conjugated secondary antibody for 1 h at room temperature, and then exposed for detection.

For the extraction of nuclear and cytosol protein from MC3T3 cells, a nuclear and cytoplasmic protein extraction kit (Beyotime, P0028) was used. The purity of separation was determined by nuclear specific marker H3. For the extraction of nucleolar protein from MC3T3 cells, nucleolar protein separation was performed following the steps outlined in the reference^40^ and verified with Coomassie blue staining (Figure S3, Supporting Information). After conducting the Western blot experiments as described above, Coomassie Blue staining was used to stain histones in the 15 kDa region as an internal control. The antibodies used in Western blotting are shown in Table S7 (Supporting Information).

### Enzyme‐Linked Immunosorbent Assay (ELISA)

For the osteocytes’ medium after Flexcell treatment, the supernatant was obtained by centrifugation. For 6‐week‐old male mice maxilla, the maxilla and skull were separated, weighed at ≈0.05 g, and cut into 0.5 mm^2^ bone slices. ≈2 µL of interstitial fluid could be obtained by centrifugation. After adding 100 µL PBS and centrifugation at 1000 rpm for 20 min, the diluted mouse maxillary/cranial interstitial fluid was obtained. Subsequently, the mouse FGF9 ELISA experiment was performed according to the manufacturer's instructions (ABclonal, RK02809) for incubation and color reaction. After obtaining the optical density (OD) values, the standard curve was fitted using the CurveExpert 1.4 software to calculate the concentration of the samples.

### Crystal Violet Staining

After osteoclast induction of BMDMs for 7 days, the cells were fixed with 4% PFA, followed by staining with crystal violet solution (Beyotime, C0121) for 10 min. DAPI was used to label the cell nuclei. Images were captured using a fluorescence microscope, and the number of fused nucleus within the osteoclasts was counted.

### Statistical Analysis

Statistical analysis was performed using the SPSS 21.0 software. Statistical bar graphs were generated using GraphPad Prism 8.0 software. Comparisons between two groups were analyzed using an independent samples *t*‐test, while paired *t*‐tests were employed for comparing the means of two samples in a grouped design. Nonparametric tests were used when the data did not meet normality assumptions. All tests were two‐tailed, with a significance threshold of α = 0.05. Data results are presented as mean ± standard deviation and the sample size (*n*) for each experiment was indicated in the corresponding figure legend or table, with *p* < 0.05 considered statistically significant (*p < 0.05; **p < 0.01; ***p < 0.001).

## Conflict of Interest

The authors declare no conflict of interest.

## Supporting information



Supporting Information

## Data Availability

The data that support the findings of this study are available from the corresponding author upon reasonable request.
