# Supplementary material for: FGF9–FGFR2 Signaling via Osteocytes‐Preosteoblasts Crosstalks to Mediate Mechanotransduction‐Driven Intramembranous Osteogenesis in the Underdeveloped Maxilla
Source: Adv Sci (Weinh). 2025 Sep 6;12(44):e06954. doi: 10.1002/advs.202506954 (PMC12667477; doi:10.1002/advs.202506954)
Supplement: Supplementary file 1 — Supporting Information [file ADVS-12-e06954-s001.docx]

**Supporting Information**

Supporting Information is available from the Wiley Online Library or from the author.

Supporting Information

**FGF9–FGFR2 Signaling via Osteocytes-Preosteoblasts crosstalks to Mediate Mechanotransduction-Driven Intramembranous Osteogenesis in the Underdeveloped Maxilla**

*Yiwen Zhou#, Lili Chen#, Miaomiao Han, Peixiang Zhu, Yanyi Wang, Xuanxuan Yu, Tingyu Reng, Huijuan Wang, Baochao Li, Caixia Zhang, Ziwei Huang, Shuang Lin, Guoyun Wang, Jie Ge, Baosheng Guo*, Huang Li**

**
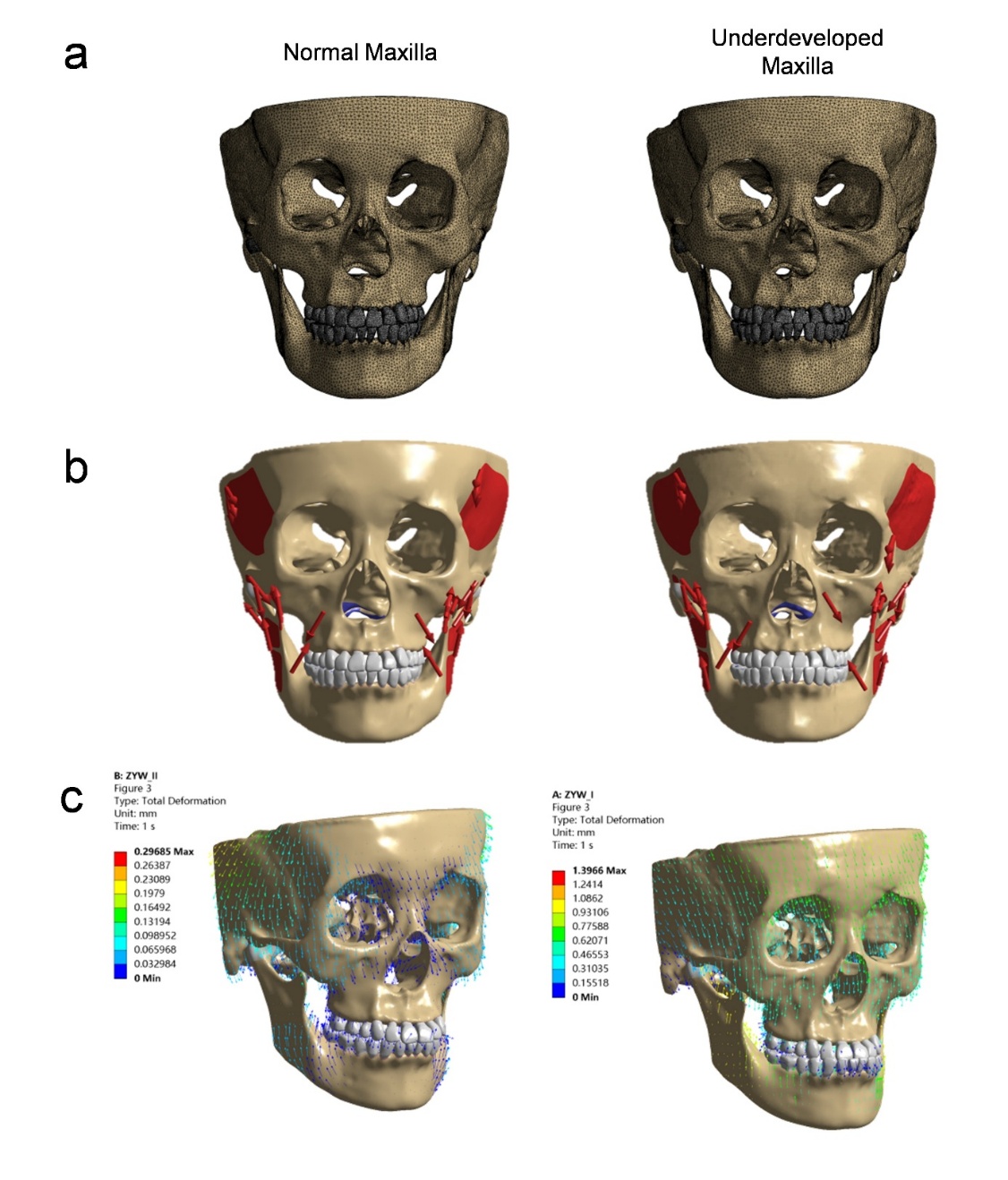
**

**Figure S1. Three-dimensional finite element analysis of cranial models with normal versus underdeveloped maxilla.** **a)** Cranial models illustrating normal and underdeveloped maxilla. **b)** Schematic representation of masticatory muscle force loading. **c)** Displacement trends of the finite element models under masticatory muscle force.


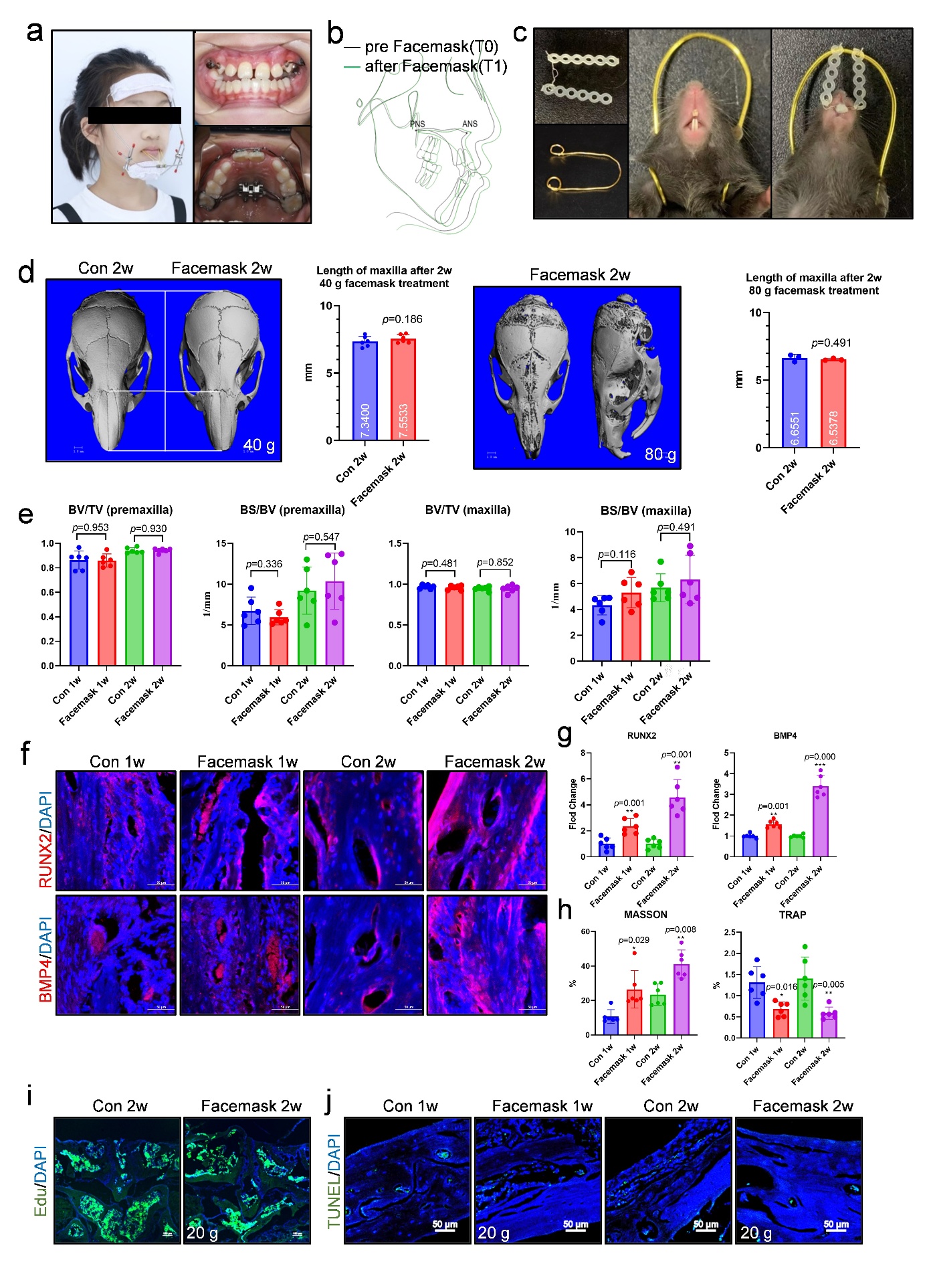


**Figure S2. Clinical and experimental analysis of maxillary facemask protraction. a)** Photograph of a patient wearing a facemask, illustrating both extraoral and intraoral components. **b)** Lateral cephalograms indicating the PNS (posterior nasal spine) and ANS (anterior nasal spine) landmarks, with the ANS-PNS distance representing the maxillary sagittal length before and after treatment. **c)** Diagram of the experimental setup for maxillary facemask protraction in mice. **d)** Comparison of maxillary responses under different forces. 40 g traction for 2 weeks resulted in frequent model detachment and no significant increase in maxillary length (n = 6), whereas 80 g traction for 2 weeks led to 50% mortality, with the surviving mice exhibiting tooth avulsion and maxillary destruction due to excessive force (n = 3). **e)** Quantification of BV/TV and BS/BV in the premaxilla and maxilla after 1 and 2 weeks 20 g facemask traction (n = 6). **f&g)** Osteogenic Marker Analysis: Immunofluorescence staining and quantification of RUNX2, BMP4, MASSON and TRAP in the maxilla of mice subjected to 20 g protraction for 1 and 2 weeks. **h)** Quantification of Masson and TRAP staining in the maxilla of mice subjected to 20 g protraction for 1 and 2 weeks. **i)** EdU staining of the maxilla following 2 weeks of 20 g protraction. **j)** TUNEL staining of the maxilla after 1 and 2 weeks of 20 g protraction. Data are presented as mean ± SD. Statistical comparison between groups was performed using an independent samples t-test. Statistical significance was determined as follows: *P* < 0.05*, *P* < 0.01 **, *P* < 0.001***.


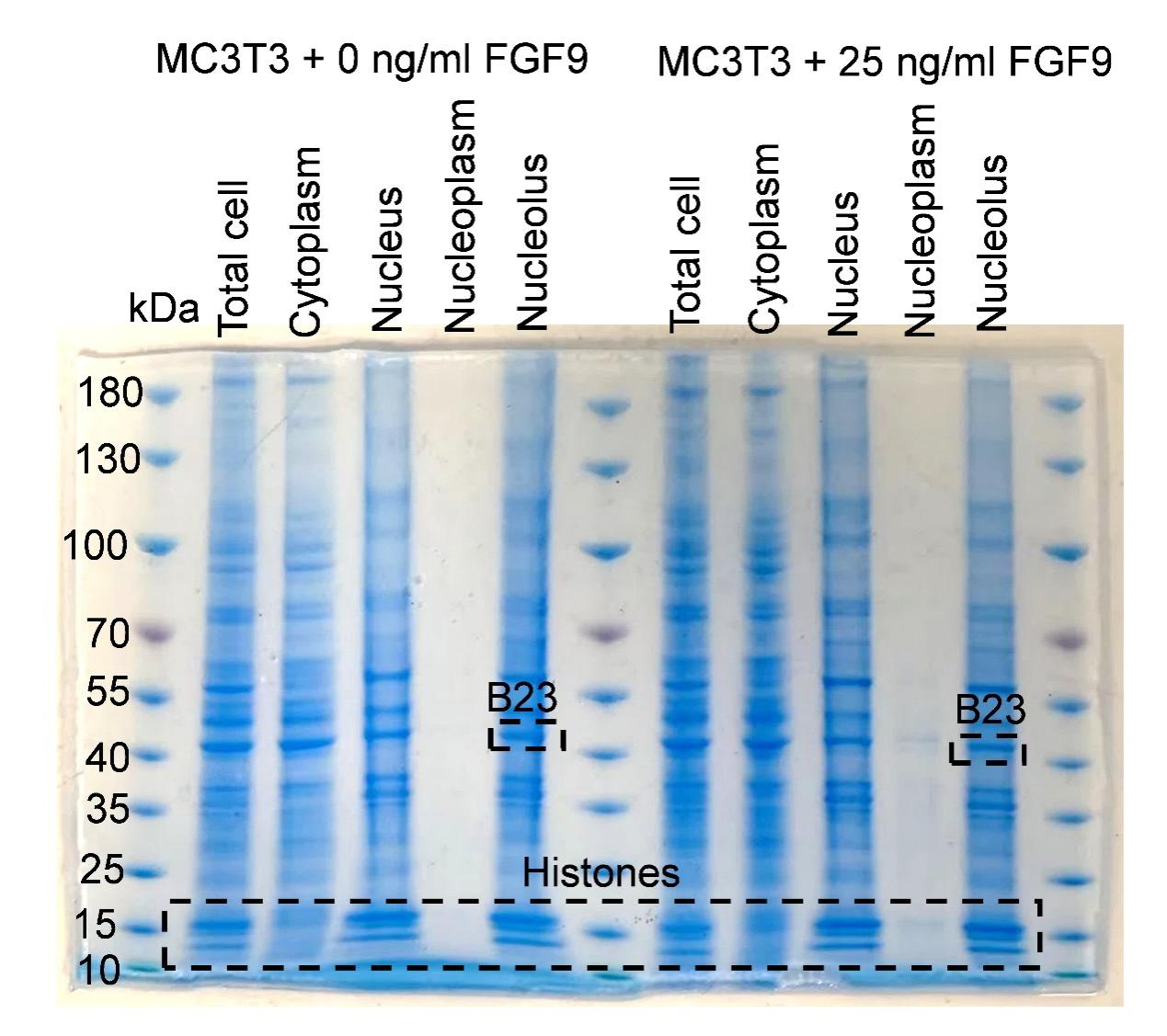


**Figure S3. Verification of nucleolar protein isolation in MC3T3 cells.** Nucleolar proteins were isolated following the established protocol, and the purity of the isolated fraction was confirmed using Coomassie Blue staining.

**
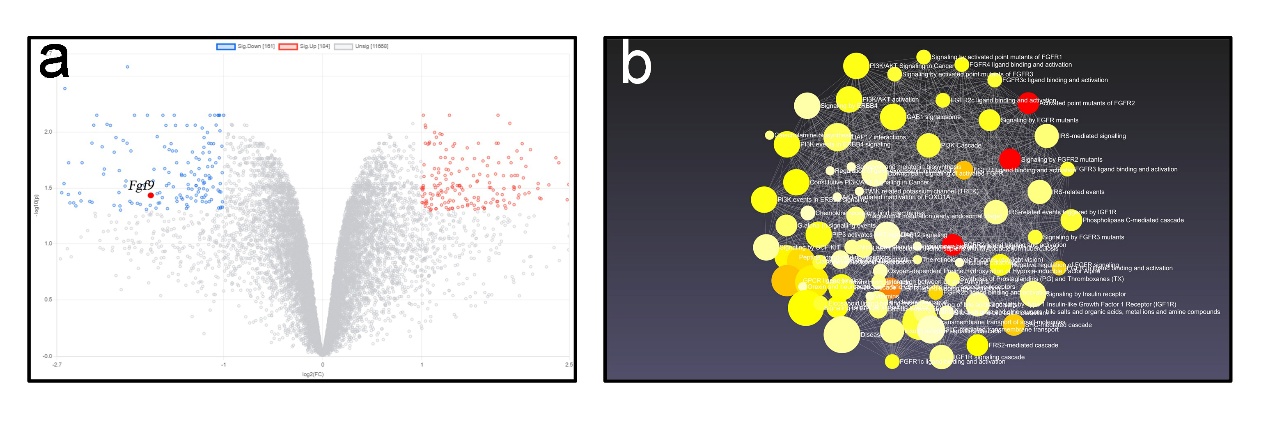
**

**Figure S4. Downregulation of FGF9 in osteocytes under fluid shear stress. a)** Volcano plot of RNA sequencing data from osteocytes exposed to fluid shear stress, highlighting differentially expressed genes. **b)** Protein interaction network analysis based on RNA sequencing data, showing FGFR2-related signaling as the most prominent pathway.


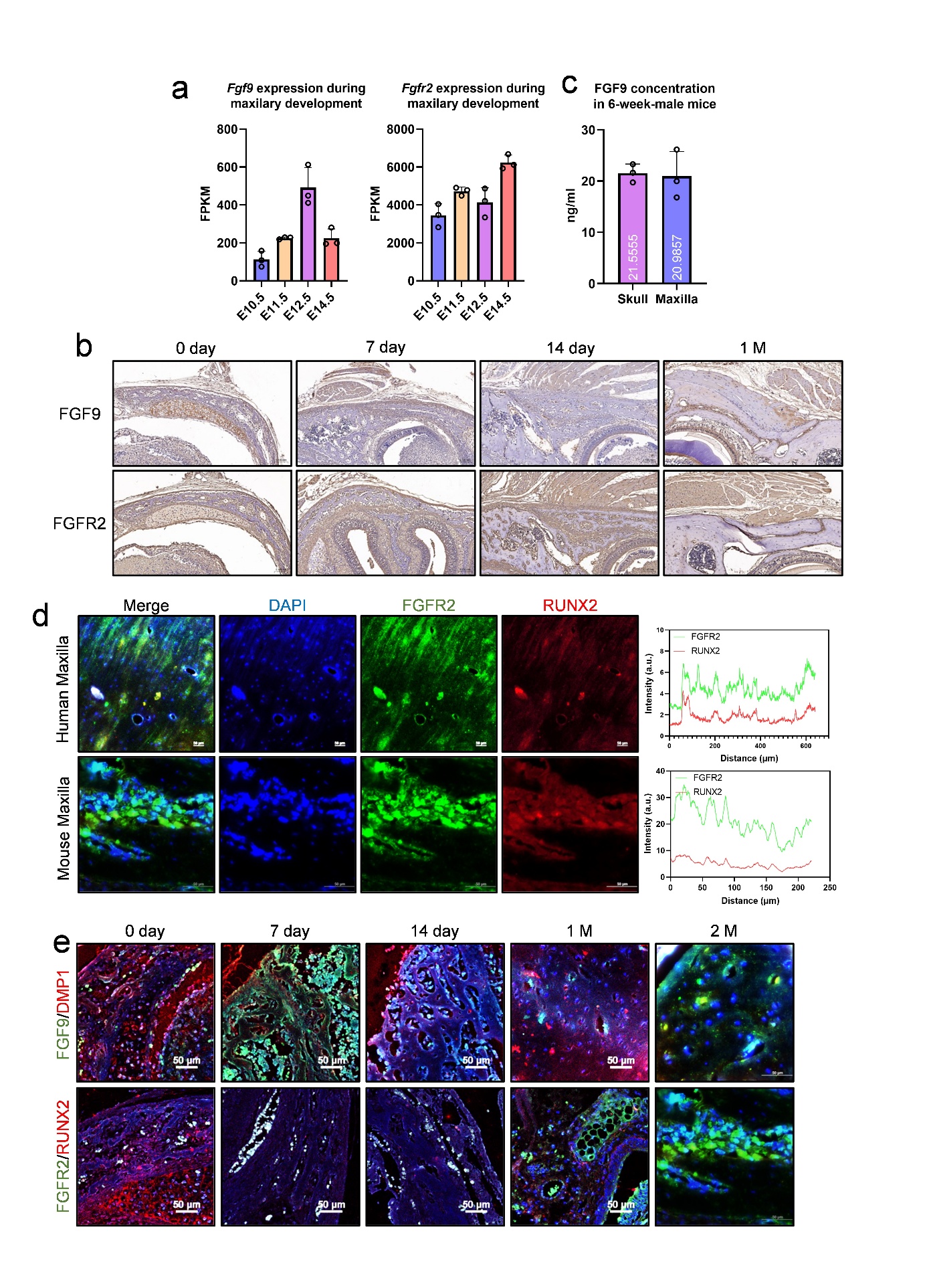


**Figure S5. Expression of FGF9 and FGFR2 during maxillary development. a)** RNA-seq analysis of *Fgf9* and *Fgfr2* expression in embryonic maxilla at E10.5, E11.5, E2.5, and E14.5 (n = 3). **b)** Immunohistochemical staining of FGF9 and FGFR2 in the mouse maxilla at postnatal day 0, 7, 14, and 1 month (n = 3). **c)** Determination of physiological FGF9 levels in the maxilla and craniofacial bones of 6-week-old mice (n = 3). **d)** Representative immunofluorescence images showing dual staining of FGFR2 and RUNX2 in human and mouse maxilla, with quantification of colocalization. **e)** Representative immunofluorescence dual staining images of FGF9 with osteocyte markers DMP1 and FGFR2 with osteoblast markers RUNX2 in the mouse maxilla at postnatal day 0, 7, 14, 1 month, and 2 months.


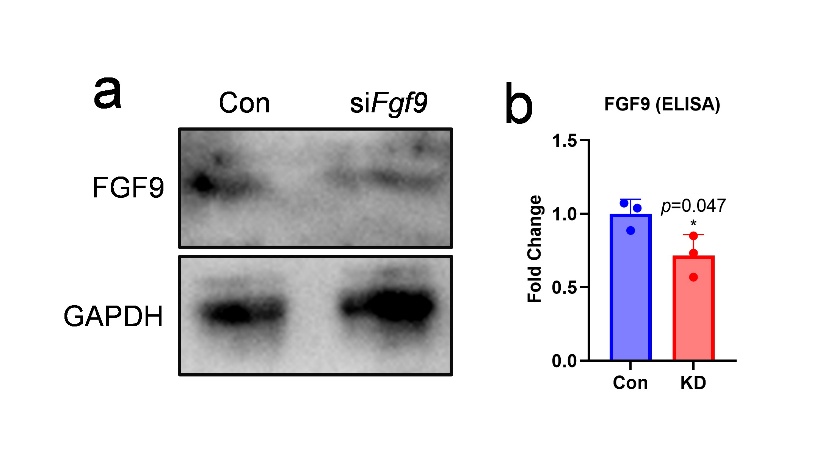


**Figure S6. Validation of siRNA-mediated *Fgf9* knockdown efficiency in osteocytes. a)** Western blot analysis 48 hours post-siRNA treatment. **b)** ELISA quantification of *Fgf9* levels in osteocyte supernatants following siRNA treatment. Data are presented as mean ± SD. n = 3. Statistical comparison between groups was performed using an independent samples t-test. Statistical significance was determined as follows: *P* < 0.05*, *P* < 0.01 **, *P* < 0.001***.


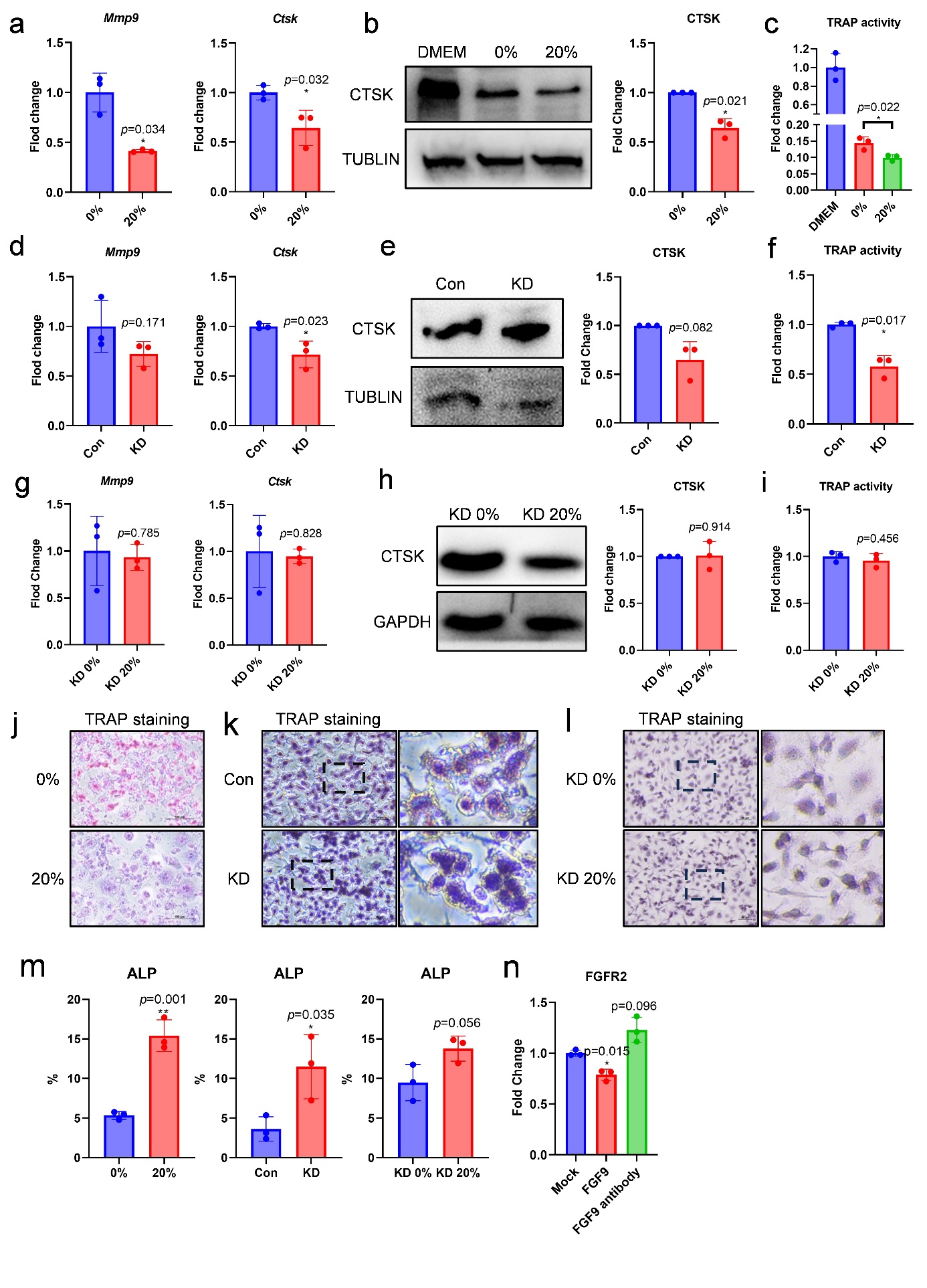


**Figure S7. Downregulation of FGF9 in osteocytes under mechanical stress promoted osteoclast differentiation.** **a–c&j)** Co-culture of osteoclasts with conditioned medium from osteocytes with mechanical stress. **(a)** mRNA expression levels of osteoclast-related markers *Mmp9* and *Ctsk*. **(b)** Western blot images and quantification of CTSK. **(c)** TRAP enzymatic activity. **(j)** Representative TRAP staining images. **d–f&k)** Co-culture of osteoclasts with conditioned medium from FGF9-knockdown osteocytes. **(d)** mRNA expression levels of osteoclast-related markers *Mmp9* and *Ctsk*. **(e)** Western blot images and quantification of CTSK. **(f)** TRAP enzymatic activity. **(k)** Representative TRAP staining images. **g–i&l)** Co-culture of osteoclasts with conditioned medium from FGF9-knockdown osteocytes treating with mechanical stress. **(g)** mRNA expression levels of osteoclast-related markers *Mmp9* and *Ctsk*. **(h)** Western blot images and quantification of CTSK. **(i)** TRAP enzymatic activity. **(l)** Representative TRAP staining images. **m)** Quantification of ALP staining in MC3T3 treated with osteocyte-conditioned medium. **n)** Quantification of FGFR2 in newborn maxilla treated with 25 ng/mL recombinant FGF9 or 1 μl/mL FGF9 neutralizing antibody. Data are presented as mean ± SD. n = 3. Statistical comparison between groups was performed using an independent samples t-test. Statistical significance was determined as follows: *P* < 0.05*, *P* < 0.01 **, *P* < 0.001***.


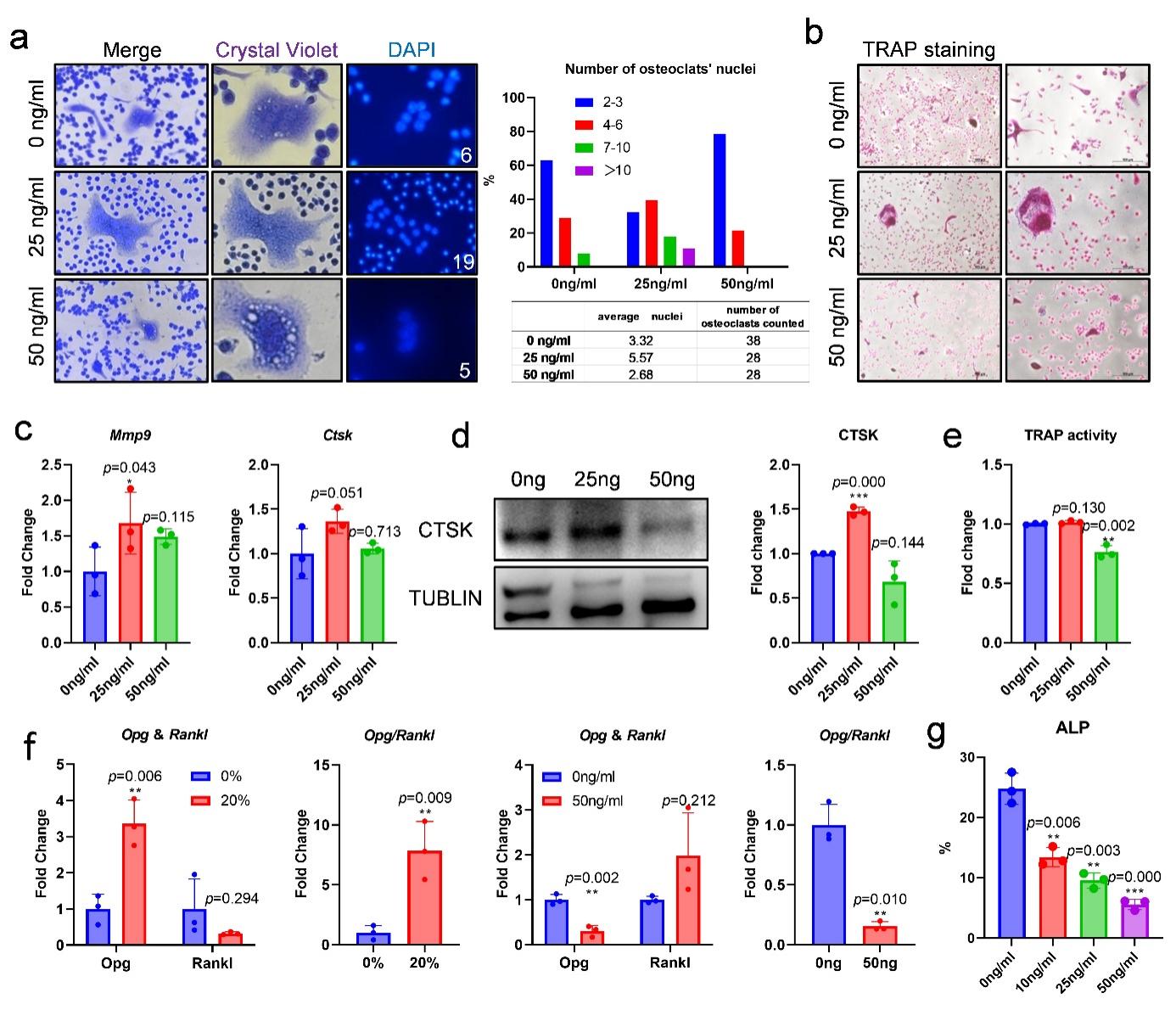


**Figure S8. 25 ng/ml FGF9 promoted osteoclasts fusion and differentiation.**

Osteoclasts following treatment with 0, 25, and 50 ng/mL FGF9: **a)** Dual staining with crystal violet and DAPI to visualize and quantify nuclear fusion. **b)** Representative TRAP staining images. **c)** mRNA expression levels of *Mmp9* and *Ctsk*. **d)** Western blot analysis and quantification of CTSK. **e)** TRAP enzymatic activity. **f)** OPG/RANKL mRNA expression in MC3T3 under osteocytes’ conditional medium (0% and 20%) or FGF9 recombinant protein (0 and 50 ng/ml). **(g)** Quantification of ALP staining in MC3T3 treated with 0-50 ng/ml FGF9 recombinant protein. Data are presented as mean ± SD. n = 3. Statistical comparison between groups was performed using an independent samples t-test. Statistical significance was determined as follows: *P* < 0.05*, *P* < 0.01 **, *P* < 0.001***.


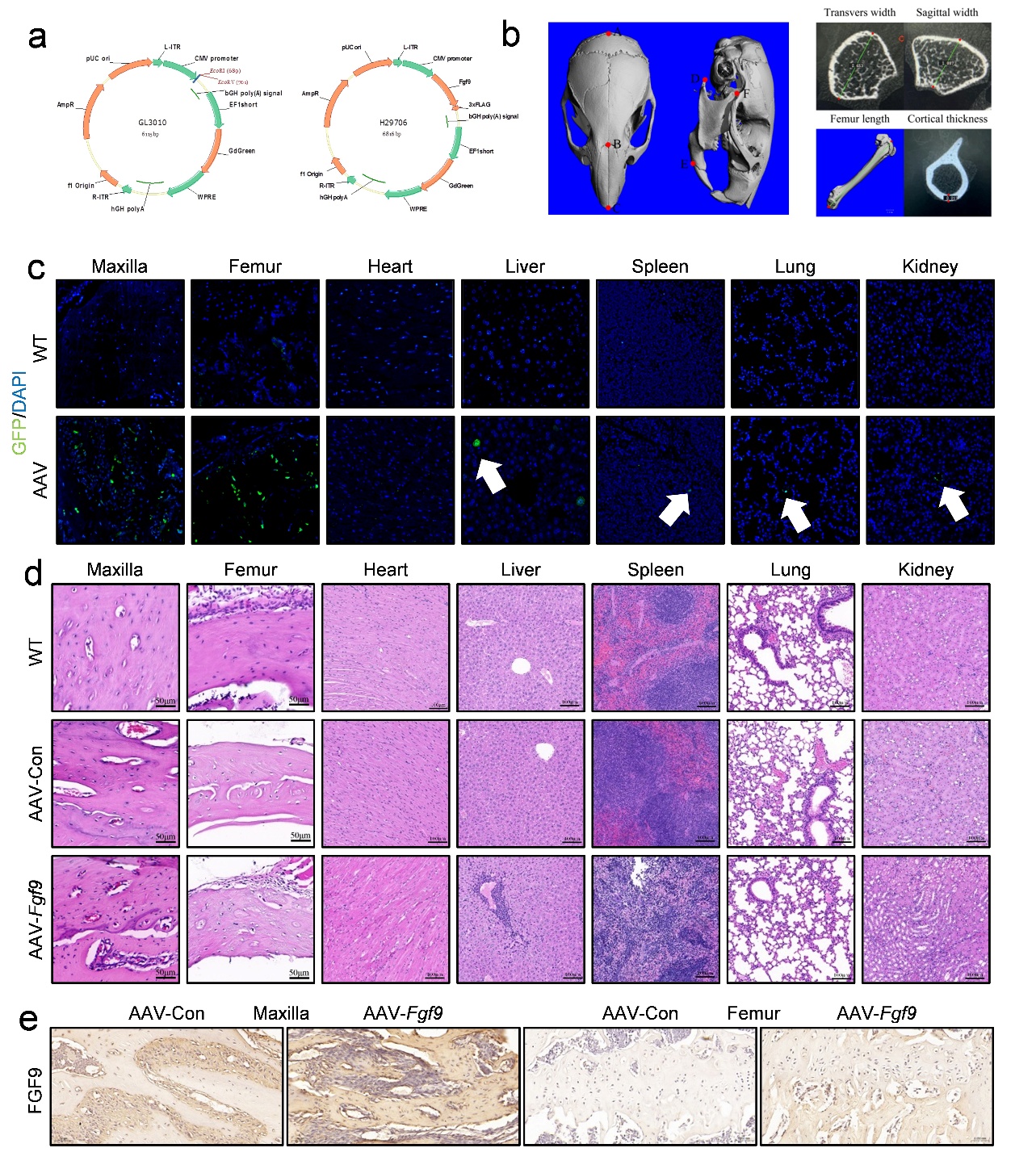


**Figure S9. Construction and validation of bone-targeted adenovirus overexpressing *Fgf9*. a)** Schematic diagram of the bone-targeted adenoviral construct for *Fgf9* overexpression (empty vector on the left, full construct on the right). **b)** Schematic illustration of the measurement parameters for craniofacial and femoral bones, including maxilla, cranium, and mandible (length and width) as well as femur (length, width, and cortical thickness): A–C: cranium length; B–C: maxilla length; D–E: mandible length; D–F: mandible width. **c)** GFP expression in the maxilla, femur, heart, liver, spleen, lung, and kidney following AAV transfection. White arrows indicate GFP-positive regions. **d)** Hematoxylin and eosin staining of the maxilla, femur, heart, liver, spleen, lung, and kidney after AAV transfection.**e)** Immunohistochemical staining for FGF9 in the maxilla and femur following AAV-*Fgf9* transfection. Data are presented as mean ± SD. n = 6. Statistical comparison between groups was performed using an independent samples t-test. Statistical significance was determined as follows: *P* < 0.05*, *P* < 0.01 **, *P* < 0.001***.


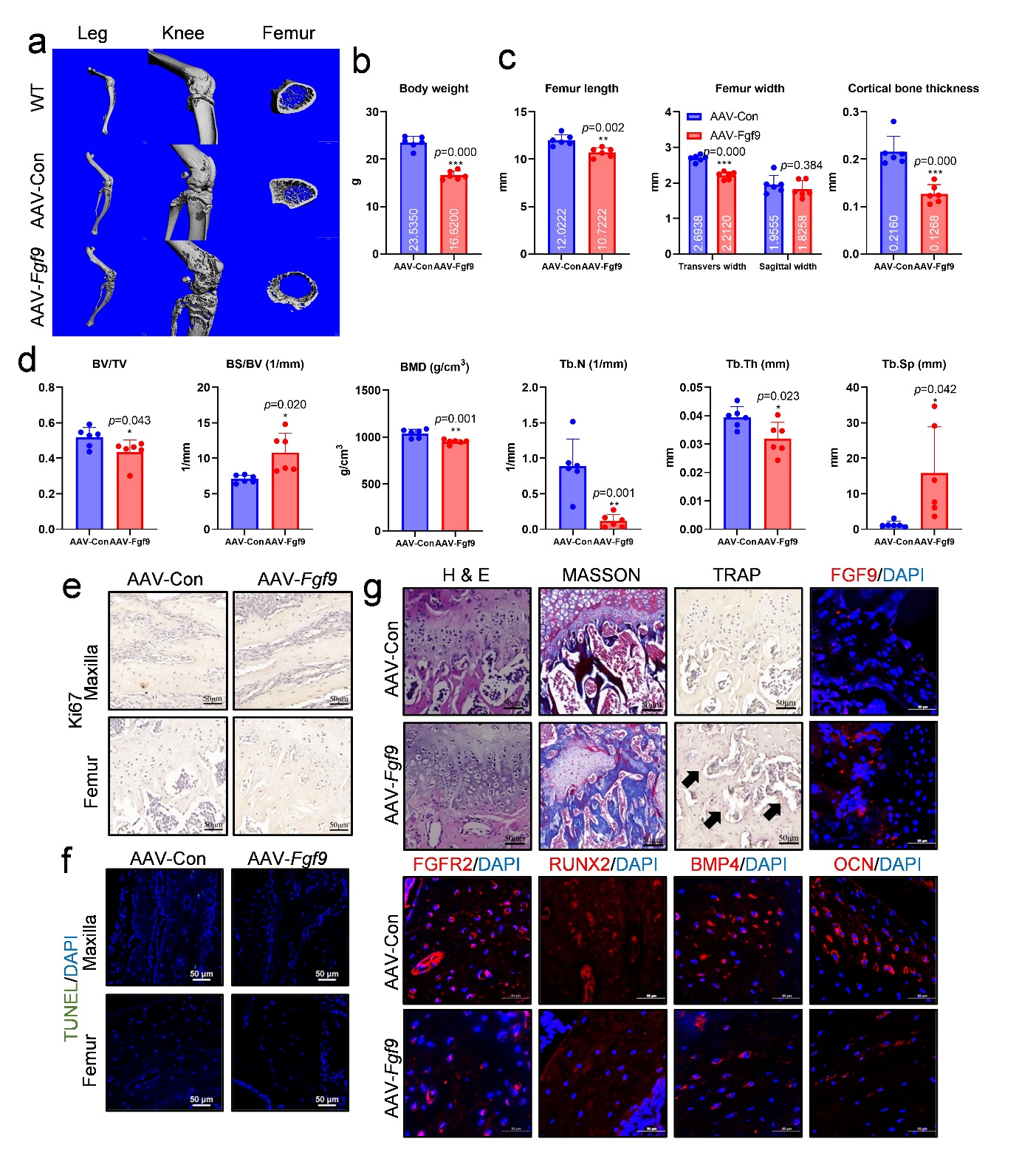


**Figure S10. Bone-targeted overexpression of FGF9 induces long bone underdevelopment in mice. a)** Three-dimensional Micro-CT reconstruction of the long bones and femoral cross-section. **b)** Body weight of mice overexpressing FGF9. **c)** Measurements of femoral length, width, and cortical thickness. **d)** Quantitative analysis of femoral diaphyseal and metaphyseal parameters: BV/TV, BS/BV, BMD, Tb.N, Tb.Th, and Tb.Sp. **e)** Representative immunohistochemical images of KI67 staining in the maxilla and femur. **f)** Representative TUNEL staining images in the maxilla and femur. **g)** Representative images of the femur showing H&E, Masson, and TRAP staining, as well as immunofluorescence for FGF9, FGFR2, RUNX2, BMP4, and OCN. Black arrows indicate TRAP-positive regions. Data are presented as mean ± SD. n = 6. Statistical comparison between groups was performed using an independent samples t-test. Statistical significance was determined as follows: *P* < 0.05*, *P* < 0.01 **, *P* < 0.001***.


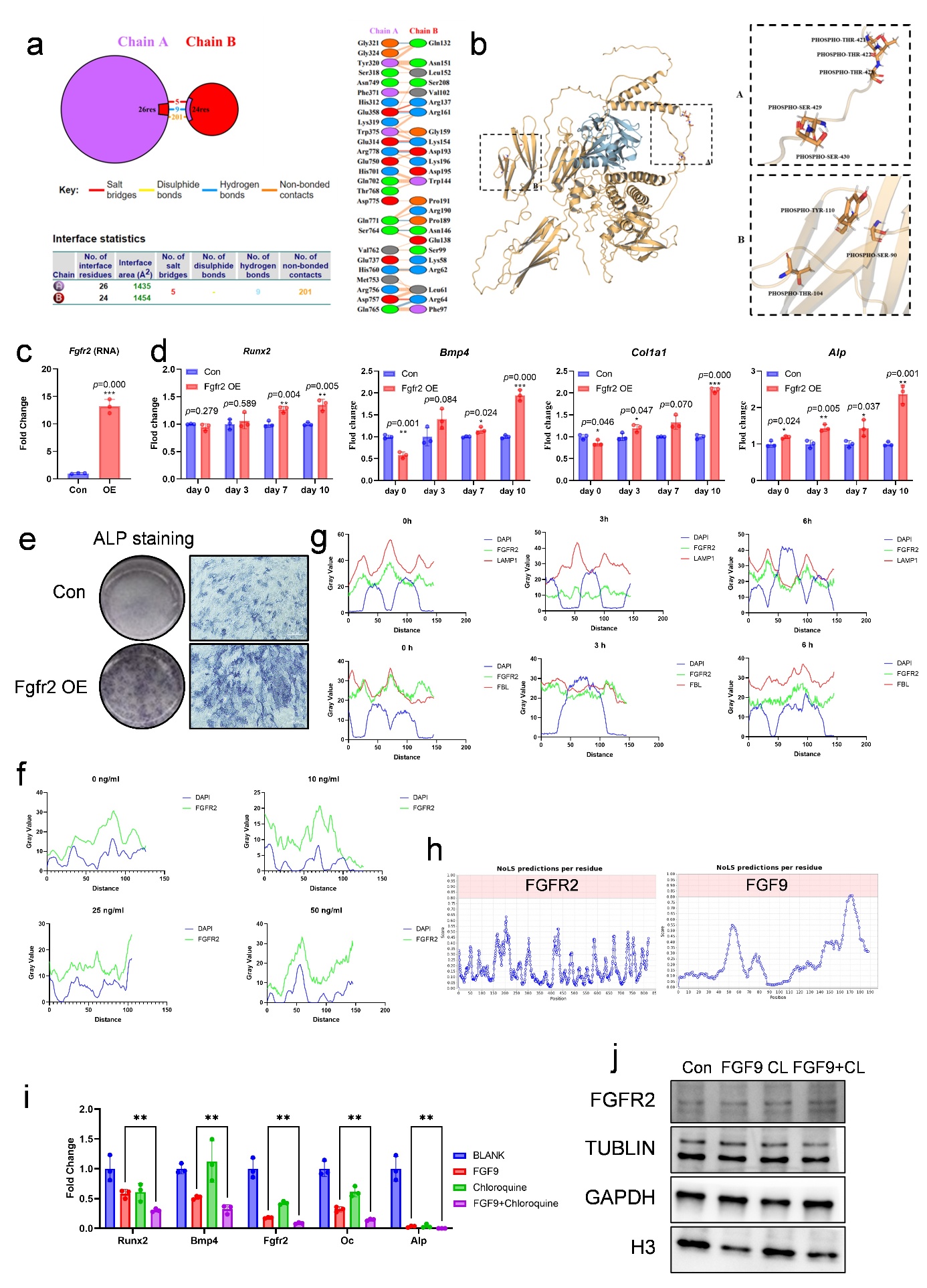


**Figure S11. High-Affinity interaction between FGF9 and the Oosteogenic regulator FGFR2 induces FGFR2 nuclear translocation via a nuclear localization sequence. a)** Predicted protein binding sites between mouse FGF9 and FGFR2. **b)** Phosphorylation sites on FGFR2 following FGF9 treatment. **c-e)** Lentivirus-mediated overexpression of *Fgfr2* in periosteal stem cells (PSCs, n = 3). **(c)** mRNA expression levels of *Fgfr2* after overexpression. **(d)** mRNA expression levels of osteogenic markers (*Runx2, Bmp4, Col1a1, Alp*) at 0, 3, 7, and 10 days of osteogenic induction. **(e)** Representative ALP staining images. **f)** Quantification of FGFR2 and DAPI co-localization in MC3T3 treated with 0-50 ng/ml concentrations of FGF9 for 24 h. **g)** Quantification of FGFR2 co-localization with LAMP1 and FBL in MC3T3 after 3 or 6 h 25 ng/ml FGF9 treatment. **h)** Predicted nuclear localization sequence in FGFR2 associated with its interaction with FGF9. **i)** mRNA expression of osteogenic markers and *Fgfr2* in MC3T3 treated with 10 μM chloroquine and/or 25 ng/mL FGF9 (n = 3). **j)** Nuclear FGFR2 protein expression in MC3T3 treated with 10 μM chloroquine and/or 25 ng/mL FGF9 (n = 2). Data are presented as mean ± SD. Statistical comparison between groups was performed using an independent samples t-test. Statistical significance was determined as follows: *P* < 0.05*, *P* < 0.01 **, *P* < 0.001***.

**Table S1. Properties of the materials considered in this study.**

|  | Material Young’s modulus (Mpa) | Poisson’s ratio |
| --- | --- | --- |
| Tooth | 1.96× 10^4^ | 0.3 |
| Cortical bone | 1.37× 10^4^ | 0.26 |
| Parodontium | 6.9× 10^−1^ | 0.49 |
| Cartilage | 7.9 × 10^−1^ | 0.49 |
| Disc | 4.41 × 10 | 0.4 |

**Table S2. Normal Masticatory Muscle Forces Added to the Model.**

| Number | Muscle Group | Force (N) |
| --- | --- | --- |
| 1 | Origin of the Masseter Muscle - Left side | 190.39 |
| 2 | Deep Part of the Masseter Muscle - Left side | 81.634 |
| 3 | Posterior Part of the Temporalis Muscle - Left side | 75.561 |
| 4 | Middle Part of the Temporalis Muscle - Left side | 95.593 |
| 5 | Anterior Part of the Temporalis Muscle - Left side | 158.02 |
| 6 | Medial Pterygoid Muscle - Left side | 174.89 |
| 7 | Origin of the Masseter Muscle - Right side | 190.39 |
| 8 | Deep Part of the Masseter Muscle - Right side | 81.634 |
| 9 | Posterior Part of the Temporalis Muscle - Right side | 75.561 |

**Table S3. Shedding rate and death rate of mouse facemask model.**

| Group (6 mice/group) | Model fall-off times | Number of dead mouse |
| --- | --- | --- |
| 20g | 2 | 0 |
| 40g | 13 | 0 |
| 80g | 18 | 3 |

**Table S4. Antibodies for immunohistochemical staining and immunofluorescence.**

| Antibodies | Dilution rate | Manufacturer |
| --- | --- | --- |
| FGF9 (A6374) | 1:200 | ABclonal, Wuhan, China |
| FGF9 (mouse anti mouse, sc-8413) | 1:100 | Santa Cruz Biotechnology, Shanghai, China |
| FGFR2 (A19051) | 1:200 | ABclonal, Wuhan, China |
| DMP1 (YN0112） | 1:200 | Immunoway, Suzhou, China |
| RUNX2 (A11753) | 1:200 | ABclonal, Wuhan, China |
| BMP4 (A11315） | 1:100 | ABclonal, Wuhan, China |
| KI67 (A20018) | 1:200 | ABclonal, Wuhan, China |
| OCN (GB11233-100) | 1:200 | Serbicebio, Wuhan, China |
| Anti-rabbit secondary antibody 488 (ab150077) | 1:1000 | Abcam, Shanghai, China |
| Anti-mouse secondary antibody 555 (sc-516177) | 1:100 | Santa Cruz Biotechnology |
| Anti-rabbit secondary antibody conjugated with HRP (GB23303) | 1:200 | Serbicebio, Wuhan, China |

**Table S5. siRNA sequence.**

| Gene | siRNA sequence (5’ to 3’) |
| --- | --- |
| *Fgf9* | UGACAAAGUACCUGAACUATT |
|  | UAGUUCAGGUACUUUGUCATT |
| *Nr2f1* | CCUCAAAGCCAUCGUGCUATT |
|  | UAGCACGAUGGCUUUGAGGTT |
| *Atf5* | GCUCGUAGACUAUGGGAAATT |
|  | UUUCCCAUAGUCUACGAGCTT |

**Table S6. Primer sequence.**

| Gene | Primer sequence (5’ to 3’) |
| --- | --- |
| *Gapdh* Forward Primer | AATGGATTTGGACGCATTGGT |
| *Gapdh* Reverse Primer | TTTGCACTGGTACGTGTTGAT |
| *Runx2* Forward Primer | GACTGTGGTTACCGTCATGGC |
| *Runx2* Reverse Primer | ACTTGGTTTTTCATAACAGCGGA |
| *Bmp4* Forward Primer | TGGACACCTCATCACACGAC |
| *Bmp4* Reverse Primer | GCCCAATCTCCACTCCCTTG |
| *Fgf9* Forward Primer | CCTTTCTGCCTGCTGAGAGTC |
| *Fgf9* Reverse Primer | CCTGCACACCGAAATAGCTC |
| *Fgfr2* Forward Primer | CGCCGTGATCAGTTGGACTA |
| *Fgfr2* Reverse Primer | GTCCAGTACGGTGCTCTCTG |
| *Oc* Forward Primer | TTCTGCTCACTCTGCTGACC |
| *Oc* Reverse Primer | GGGACTGAGGCTCCAAGGTA |
| *Alp* Forward Primer | CATGCCCAGTGCCTTCTGATT |
| *Alp* Reverse Primer | TGAGATTCGTCCCTCCGCTGG |
| *Trap* Forward Primer | GCTGGAAACCATGATCACCT |
| *Trap* Reverse Primer | TTGAGCCAGGACAGCTGAGT |
| *Mmp9* Forward Primer | AGCCAACTATGACCAGGAT |
| *Mmp9* Reverse Primer | TGCCGTCTATGTCGTCTTTA |
| *Ctsk* Forward Primer | CTGGAGGGCCAACTCAAGAAGAAAAC |
| *Ctsk* Reverse Primer | GCCTTTGCCGTGGCGTTATACATAC |
| *Opg* Forward Primer | TGAGGTTTCCAGAGGACCAC |
| *Opg* Reverse Primer | GGAAAGGTTTCCTGGGTTGT |
| *Rankl* Forward Primer | GCTCACCTCACCATCAATGCT |
| *Rankl* Reverse Primer | GGTACCAAGAGGACAGACTGACTTTA |
| *Nr2f1* Forward Primer | TCAGATGGGTAATGAATCCAGTGT |
| *Nr2f1* Reverse Primer | TGCATACTGGCCTGGATTGG |
| *Atf5* Forward Primer | CCAATTGTTGGTGCAGCCTC |
| *Atf5* Reverse Primer | CTTCTTTTGCTTGCGGTCCC |

**Table S7. Antibodies for Western blotting.**

| Antibodies | Dilution rate | Manufacturer |
| --- | --- | --- |
| FGF9 (A6374) | 1:1000 | ABclonal, Wuhan, China |
| FGFR2 (A19051) | 1:1000 | ABclonal, Wuhan, China |
| RUNX2 (A11753) | 1:1000 | ABclonal, Wuhan, China |
| GAPDH (AC035） | 1:5000 | ABclonal, Wuhan, China |
| CTSK (A1782) | 1:1000 | ABclonal, Wuhan, China |
| Anti-rabbit secondary antibody conjugated with HRP (GB23303) | 1:5000 | Serbicebio, Wuhan, China |

**Table S8. Three-Dimensional Analysis of Forces in the Maxillary Tuberosity Region** **Under Normal Masticatory Muscle Forces.**

| Maxillary Tuberosity | Normal Maxilla | Undeveloped Maxilla |
| --- | --- | --- |
| X axis (N) | -1.1535e-008 | -3.7611e-008 |
| Y axis (N) | -3.4878e-007 | 1.4411e-007 |
| Z axis (N) | 6.8024e-008 | -1.8571e-007 |
| Total (N) | 3.5554e-007 | 2.3805e-007↓ |

**Table S9. Baseline data of adolescents with or without facemask tractions.**

|  | | Un-facemask-treated （n=20） | Facemask-treated （n=18） |
| --- | --- | --- | --- |
| Gender | Male | 9 | 6 |
|  | Female | 11 | 12 |
| Age | Mean | 12.1 | 9.7 |
|  | Median | 12 | 9.5 |
|  | Standard deviation | 1.68 | 1.68 |

**Table S10. Baseline data of human maxilla samples.**

|  | | Class I （n=11） | Class III （n=25） |
| --- | --- | --- | --- |
| Gender | Male | 7 | 9 |
|  | Female | 4 | 16 |
| Age | Mean | 16.45 | 21.72 |
|  | Median | 13 | 21 |
|  | Standard deviation | 8.09 | 3.35 |
